# Elucidating the impact of parthanatos-related microRNAs on the tumoral immune microenvironment and clinical outcome in low-grade gliomas

**DOI:** 10.1007/s12672-024-01025-w

**Published:** 2024-05-10

**Authors:** Penglei Zhu, Hao Wu, Buyi Zheng, Hua Wang, Yi Zou

**Affiliations:** 1 Department of Neurosurgery, Wenzhou People’s Hospital, No.299, Gushan Road, Ouhai District, Wenzhou, 325000 Zhejiang China; 2 Department of Neurosurgery, The First Affiliated Hospital of Wenzhou Medical University, Nanbaixiang Street, Ouhai District, Wenzhou City, 325000 Zhejiang China

**Keywords:** Low-grade glioma, Parthanatos, microRNA, Prognostic model, Immune microenvironment, Personalized therapy

## Abstract

Parthanatos, a cell death mechanism triggered by PARP-1 activation, is implicated in oncogenic processes, yet their role in low-grade gliomas (LGG) remains poorly understood. This research investigates Parthanatos-related miRNAs' prognostic and immunomodulatory potential, alongside their influence on therapeutic outcomes in LGGs. Comprehensive miRNA and mRNA profiles of LGG patients were extracted from TCGA and CGGA databases, integrating clinical parameters to identify Parthanatos-associated miRNAs. IHC data validated the expression levels of Parthanatos-related genes in glioma versus normal brain tissues. Protein–protein interaction networks and Spearman correlation analysis facilitated the identification of key miRNAs. Parthanatos-related miRNA indices (PMI) were screened using Lasso and assessed for their accuracy in predicting prognosis, comparing their associated potential molecular functions and heterogeneity of the immune microenvironment. Drug sensitivity was assessed between different groups and optimal therapeutic agents were predicted. Validate the expression levels of key miRNAs by qPCR. Ninety-one miRNAs significantly associated with Parthanatos were screened, through which a PMI prognosis model of nine miRNAs was constructed. The PMI score was able to independently predict the prognosis of patients with LGG, and the nomogram constructed based on the PMI provided a practical tool for clinical prediction of patient prognosis. The proportion of immune response was lower in patients in the high-risk group, and there were significant differences in drug sensitivity between different risk classes, while drugs such as Fasudil were identified as the most promising therapeutic agents for patients in the high-risk group. Our findings highlight the critical role of Parthanatos-associated miRNAs in the progression and treatment of LGG, offering novel insights into their prognostic value and therapeutic potential.

**Supplementary Information:**

The online version contains supplementary material available at 10.1007/s12672-024-01025-w.

## Introduction

In adults, gliomas represent the predominant form of primary brain tumors [1–3], with Low-grade gliomas (LGGs) being distinguished by their less aggressive nature [4]. Although LGGs typically have a better initial treatment response and relatively longer survival than higher-grade glioma (HGG), patients rarely achieve a complete cure [5]. Over time, patients with LGG are often at risk of inevitable recurrence and progression to HGG, and survival remains a concern [1, 5]. Moreover, during clinical treatment, therapeutic choices are often influenced by the molecular characteristics of the tumour, such as IDH mutation status, 1p19q co-deletion status, and other genetic variants [3, 6].

In recent years, therapies based on programmed cell death have brought a new light to the treatment of LGG [7, 8], among which Parthanatos, as a mechanism of programmed cell death, has attracted much attention in oncological research [9]. In sharp contrast to conventional apoptosis, necrosis, or autophagy, Parthanatos promotes cell death primarily through the release of apoptosis-inducing factor (AIF) and hyperactivation of poly (adenylate ribose) polymerase 1 (PARP-1) [9–11]. Its unique multistage cascade death pathways: PARP1, PARG, ARH3, AIF and MIF, etc., also provide novel targets for disease treatment [9]. In addition to this, Parthanatos has also been found to be involved in the pathogenesis of neurological disorders including glioma and neurodegenerative diseases [12], and induction of Parthanatos is expected to be a promising therapeutic strategy.

MicroRNAs (miRNAs) play a crucial regulatory role in the pathological process of LGG [13, 14]. As a class of small-molecule non-coding RNAs, miRNAs intricately regulate the expression of oncogenes and oncogenes in tumors by inhibiting the translation of specific mRNAs or promoting their degradation [15]. Although the function of miRNAs in a variety of tumors has been extensively studied [16, 17], their role in the Parthanatos pathway and how this role affects the prognosis, immune response and therapeutic efficacy of patients with LGG is still not fully understood. Unraveling the roles of Parthanatos-associated miRNAs in LGG presents a gateway to decoding the disease's pathogenesis, guiding therapy choices, and forecasting treatment success.

This investigation targets the effects of Parthanatos-related miRNAs on LGG prognoses. We melded clinical insights with high-throughput sequencing, devising a miRNA-based prognostic model to scrutinize their expression across LGG varieties. Our probe extends into how these miRNAs intertwine with the tumor’s immune backdrop, examining their role in tweaking treatment receptivity. In uncovering these miRNAs' biological essence, we’re on a quest to pinpoint groundbreaking biomarkers therapeutic strategies, and custom-fitting LGG management.

## Methods

### Acquisition and handling of data

The Cancer Genome Atlas Program (TCGA, https://portal.gdc.cancer.gov/) was used to retrieve miRNA and mRNA datasets for LGG. MiRNA expression profiles and clinical data for 198 cases were retrieved from the Chinese Glioma Genome Atlas (CGGA). A total of 423 miRNAs, present in both datasets, were compiled for subsequent analysis (see Supplementary Fig. 1). Parthanatos-related genes (PRGs) were sourced from GeneCards (https://www.genecards.org/), selecting those with a Relevance score greater than 1. Clinical datasets underwent cleansing and the details of clinical information is detailed in Supplementary Table 1, Supplementary Table 2. They were later annotated and visualized using R packages such as ggplot2, ggalluvia, and ComplexHeatmap to display information on clinical characteristics.

### IHC

PRG protein levels in tumour and normal tissues were obtained by IHC staining experiments, and differences in the intensity of IHC staining for PRGs were confirmed by searching for and extracting PRGs protein expression profiles in gliomas and normal cerebral cortex from the Human Protein Atlas (HPA) database.

### Screening for protein–protein interactions (PPIs) and miRNAs associated with PRGs

Utilizing the STRING database (https://string-db.org/), the PPI network was established.PRGs-associated expression matrices were extracted and the correlation between PRGs expression matrix and miRNA-TCGA expression matrix was calculated using Spearman correlation analysis. The correlation was filtered by Cor > 0.3, P < 0.05, and at least 2 PRGs and 2 miRNAs were correlated, and the R package "heatmap" was used to perform the correlation heatmap visualisation, and the network was visualised using Cytoscape (Version: 3.10.1). visualisation using Cytoscape (Version: 3.10.1).

### Clustering analysis using non-negative matrix factorization (NMF)

To gain insight into the role of PRGs in tumour heterogeneity, expression matrices of miRNAs associated with PRGs were extracted and consensus clustering was performed using the NMF algorithm and the R package NMF to explore the interactions between the associated miRNAs. k-values were set in the range of 2 to 5, and the optimal k-value was determined to be 2 by referring to the classification parameter map. the R package limma was used to calculate the difference between two molecular subtypes of the differential genes (|log(FC)|> 0.585, adj P < 1). The expression heatmap of the differential genes was plotted using the R package heatmap after line log2(value + 1) transformation.

### Parthanatos-related miRNA index (PMI) modelling

Investigating the influence of 45 miRNAs closely linked to PRGs on the prognosis of LGG patients, we conducted proportional risk hypothesis tests via the R package "survival." Significant associations between miRNAs and patient overall survival were pinpointed through univariate Cox analysis (P < 0.05). This step was succeeded by the application of LASSO regression to refine and reduce the set of miRNAs holding prognostic significance. The "glmnet" R package facilitated the LASSO analysis, adept at managing potential issues of high dimensionality and multicollinearity by imposing a penalty that zeroes out certain regression coefficients, hence focusing on miRNAs with substantive effects on patient outcomes.

Each patient’s Risk score was computed using the formula: ${\text{Risk}} {\text{score}} = \sum {\left( {{\text{Exp}}\_{\text{miRNAi}} \times {\text{Coef}}\_{\text{miRNAi}}} \right)}$. Here, Exp_miRNAi denotes the expression level of each miRNA, and Coef_miRNAi is its respective regression coefficient. Utilizing this model to segregate patients into high-risk and low-risk categories, we evaluated the model's prognostic prediction power through Kaplan–Meier survival plots and ROC curves. Additionally, risk factor plots created with the "ggplot2" R package illustrated the relationship between Risk score and miRNA expression. The predictive model's reliability was further confirmed by external validation using the CGGA dataset.

### Nomogram

To assess the independent prognostic value of PMI, univariate and multivariate Cox regression analyses were performed by combining PMI scores with patients' clinical characteristics, and prognostic nomograms were constructed in the LGG patient cohort using the "rms" R package, aiming at predicting 1-, 3- and 5-year overall survival. The predictive performance of the column charts was assessed by calibration curve and decision curve analysis (DCA) to ensure the accuracy and clinical value of the model.

### Functional enrichment analysis

Differential genes in different risk groups were identified and functional enrichment analysis was performed using the clusterProfiler package. In addition, Kyoto genes and Genome Encyclopaedia (KEGG) pathways were explored in depth between the different groups using Gene Set Enrichment Analysis (GSEA), with Normalised Enrichment Score (NES) > 1 and P-value < 0.05 as the screening criterion, to identify pathways that were significantly associated with disease progression.

### Immunotherapy relevance

In this study, ImmuCellAI was used to assess immune cell infiltration and immune response status in patients with LGG, to estimate the proportion of 24 immunisations and to predict the response to treatment with immune checkpoint inhibitors in patients. Correlations between immune parameters and RiskScore were compared using Spearman correlation analysis, and correlation scatter plots were drawn by gglot2.

### Prediction of clinical therapeutic agents

The response of common chemotherapeutic, targeted and immunotherapeutic agents to treatment in different risk groups was evaluated using the R packages "pRRophetic" and "OncoPredict", and the half inhibitory concentration (IC50) was calculated to quantify drug sensitivity. Subsequently, for the top 150 differential genes in the high- and low-risk groups, the Connectivity Map (CMap) database was used to predict possible molecularly targeted drugs, and the xSum method was used to calculate the CMap score [18], thus identifying the top 5 most promising therapeutic agents.

### Cell culture and qPCR

Human neural astrocytes (No. CP-H122) and brain glioma cells (T98G (No. CL-0583) and HS 683 (CL-0362)) were purchased from Prosperity Life Sciences Ltd (Wuhan, China). All were cultured in the corresponding complete medium (CM-H122, CM-0583, CM–0362) and 95%; CO2, 5% environment. Assays for the extraction of total RNA and reverse transcription of first strand cDNA were performed using TRIzol reagent (No. 10296010, Invitrogen, Carlsbad, CA, USA). PrimeScript RT reagent Kits were used to generate first strand cDNA using Takara Bio RR047A. qPCR was performed using TB Green Premix Ex Taq II (No. RR820A, Takara Bio, Shiga, Japan) on a Touch Real-Time PCR Detection System (No. CFX96, Bio-rad, Hercules, CA, USA). The miRNA expression levels were normalised using U6 as an internal reference gene. Specific primer design and synthesis were performed by Ribo Biotech (Shanghai, China). Relative expression was calculated using the 2^(-ΔΔCt) method. All experiments were repeated three times and differences between groups were calculated using one-way ANOVA.

## Results

### Expression profiles of PRGs in LGGs and their significantly associated miRNAs

**Fig. 1:**
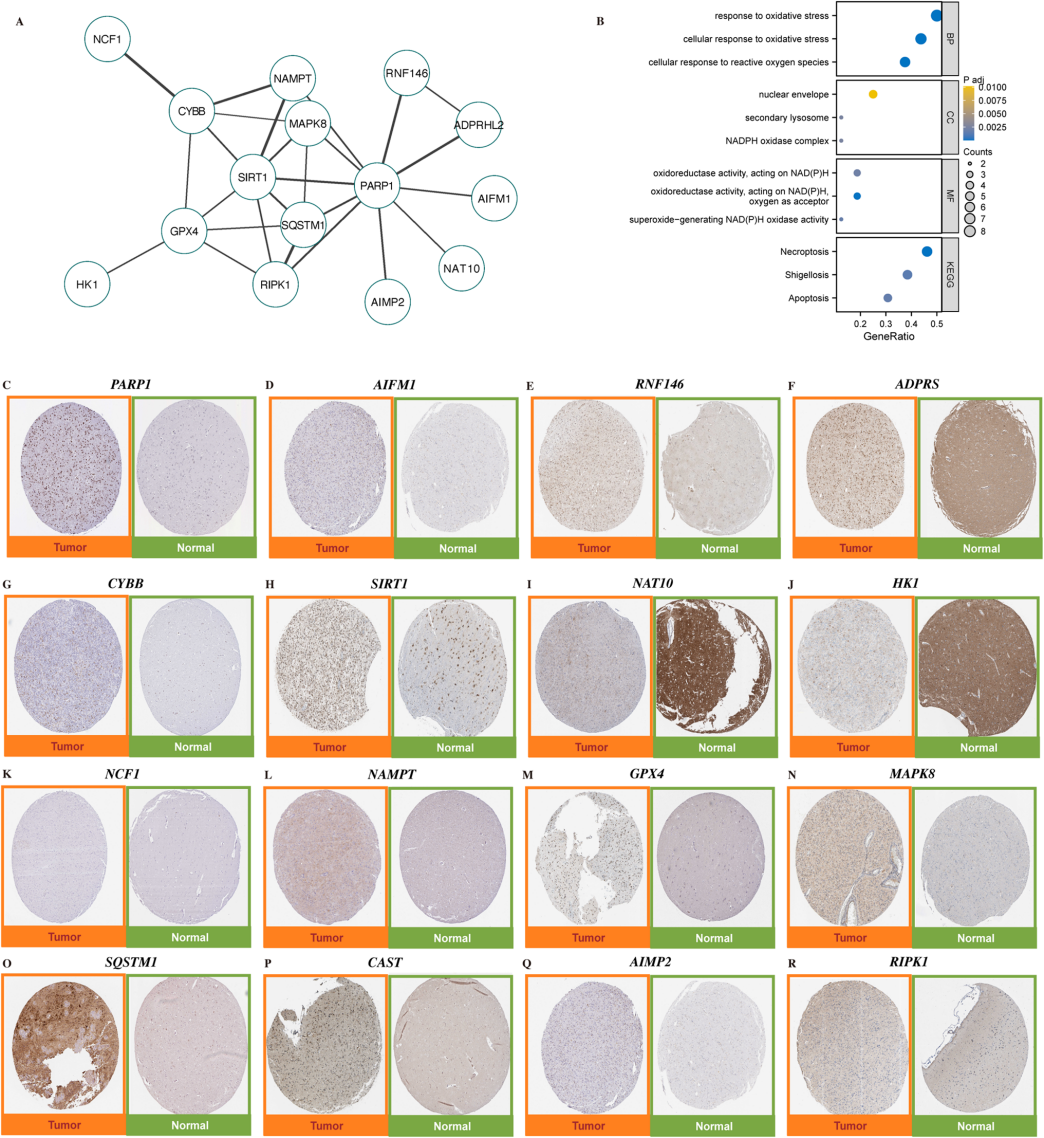
Overview of 16 Parthanatos-Related Genes (PRGs). **A** PPI network for the 16 PRGs; **B** Enrichment analysis of the 16 PRGs conducted using the Human Protein Atlas; Expression of (**C**) PARP1; **D** AIFM1; **E** RNF146; **F** ADPRS; **G** CYBB; **H** SIRT1; **I** NAT10; **J** HK1; **K** NCF1; **L** NAMPT; **M** GPX4; **N** MAPK8; **O** SQSTM1; **P** CAST; **Q** AIMP2; **R** RIPK1

**Fig. 2:**
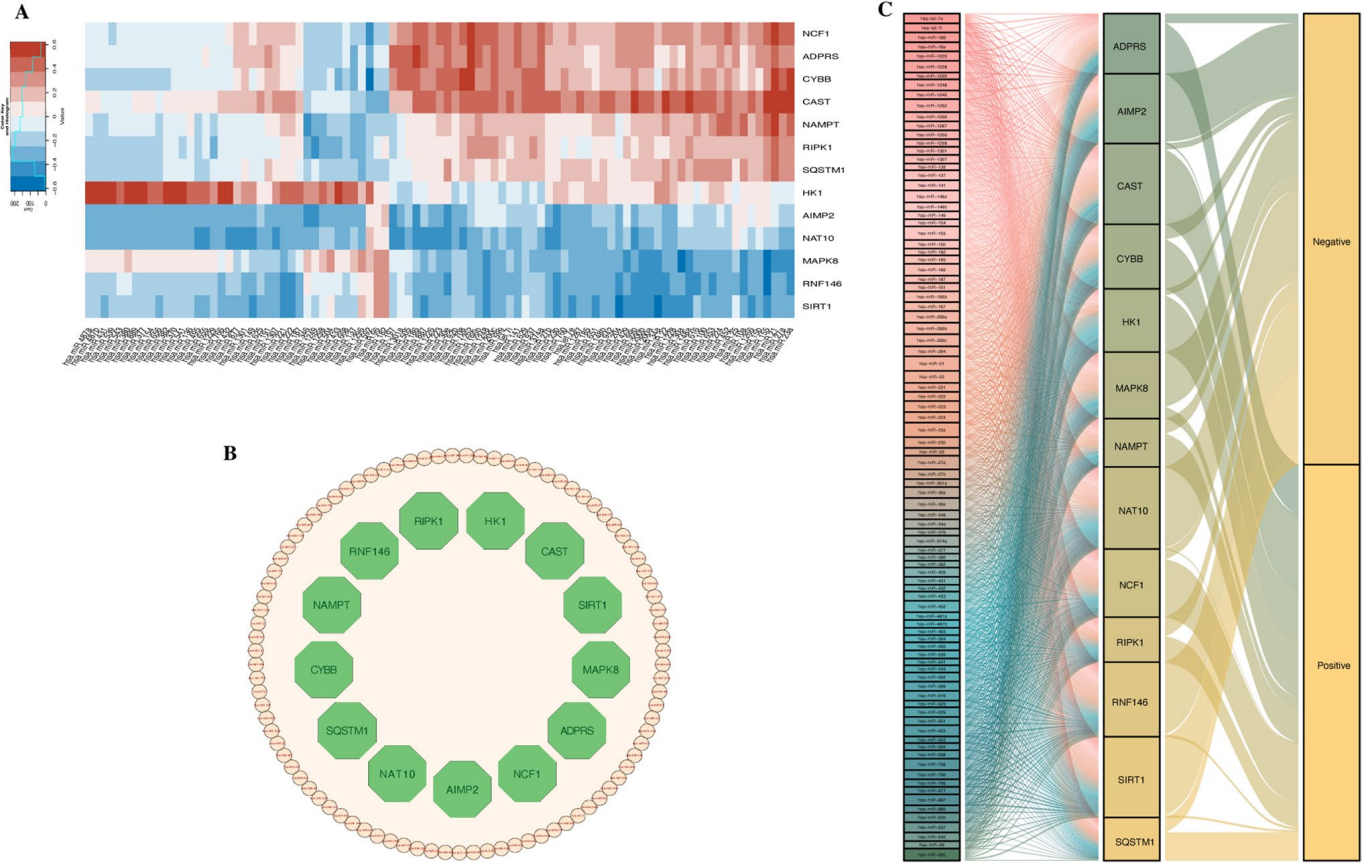
miRNAs associated with PRGs in LGGs. **A** Correlation heatmap of significantly associated PRGs and miRNAs, red represents positive correlation, blue represents negative correlation, and the shade of the color indicates the strength of the correlation; the darker the color, the stronger the correlation; **B** Network of miRNA and PRGs interactions, green circles represent PRGs, yellow diamonds represent miRNAs, and the thickness of the connecting lines indicates the strength of the correlation; **C** Sankey diagram of the regulatory relationship between miRNAs and PRGs, from PRGs to miRNAs to classification, the larger the width of the stream, the stronger the regulation

The main flow chart of this study is shown in Supplementary Fig. 2. As shown in Table 1, 16 PRGs were screened and borrowed to import the 16 PRGs into the STRING database to obtain the PPIs of PRGs (Fig. 1A), which were mainly enriched in terms of biological processes in response to oxidative stress, and were highly correlated with the signalling pathways, such as Necroptosis, Apoptosis, etc. (Fig. 1B). The expression of PRGs in tumour tissues and normal tissues was observed by IHC staining results (Fig. 1C–R). Spearman correlation analysis screened to obtain 91 miRNAs significantly correlated with PRGs (Fig. 2A), and constructed a network diagram with the relationship between PRGs and miRNAs (Fig. 2B). As a result, it was found that there were three PRGs (PARP1, AIFM1, and GPX4) associated with more than two miRNAs missing strong correlations and were left out of the network. In addition to this, PRGs exhibited complex regulatory relationships with these miRNAs (Fig. 2C).

**Table 1:** Detailed list of 16 Parthanatos related genes

Gene Symbol	Description	ENSENMBLE	Category	Uniprot ID	Gifts	GC Id	Relevance score
PARP1	Poly(ADP-Ribose) Polymerase 1	ENSG00000143799	Protein Coding	P09874	59	GC01M226360	6.915439129
AIFM1	Apoptosis Inducing Factor Mitochondria Associated 1	ENSG00000156709	Protein Coding	O95831	58	GC0XM130129	5.982010365
RNF146	Ring Finger Protein 146	ENSG00000118518	Protein Coding	Q9NTX7	44	GC06P127266	4.045446873
ADPRS	ADP-Ribosylserine Hydrolase	ENSG00000116863	Protein Coding	Q9NX46	44	GC01P037828	3.038081408
CYBB	Cytochrome B-245 Beta Chain	ENSG00000165168	Protein Coding	P04839	57	GC0XP037780	2.915173531
SIRT1	Sirtuin 1	ENSG00000096717	Protein Coding	Q96EB6	57	GC10P067884	2.111518621
NAT10	N-Acetyltransferase 10	ENSG00000135372	Protein Coding	Q9H0A0	47	GC11P034105	2.111518621
HK1	Hexokinase 1	ENSG00000156515	Protein Coding	P19367	58	GC10P069269	2.005834579
NCF1	Neutrophil Cytosolic Factor 1	ENSG00000158517	Protein Coding	P14598	55	GC07P084363	2.005834579
NAMPT	Nicotinamide Phosphoribosyltransferase	ENSG00000105835	Protein Coding	P43490	56	GC07M106248	1.868104577
GPX4	Glutathione Peroxidase 4	ENSG00000167468	Protein Coding	P36969	56	GC19P001103	1.770714641
MAPK8	Mitogen-Activated Protein Kinase 8	ENSG00000107643	Protein Coding	P45983	58	GC10P048306	1.646419287
SQSTM1	Sequestosome 1	ENSG00000161011	Protein Coding	Q13501	58	GC05P179806	1.600274324
CAST	Calpastatin	ENSG00000153113	Protein Coding	P20810	53	GC05P095975	1.494590282
AIMP2	Aminoacyl TRNA Synthetase Complex Interacting Multifunctional Protein 2	ENSG00000106305	Protein Coding	Q13155	52	GC07P010161	1.448650122
RIPK1	Receptor Interacting Serine/Threonine Kinase 1	ENSG00000137275	Protein Coding	Q13546	58	GC06P005992	1.356860161

### Identification of miRNA subtypes in LGG and differential expression of miRNAs in tumour subtypes

**Fig. 3:**
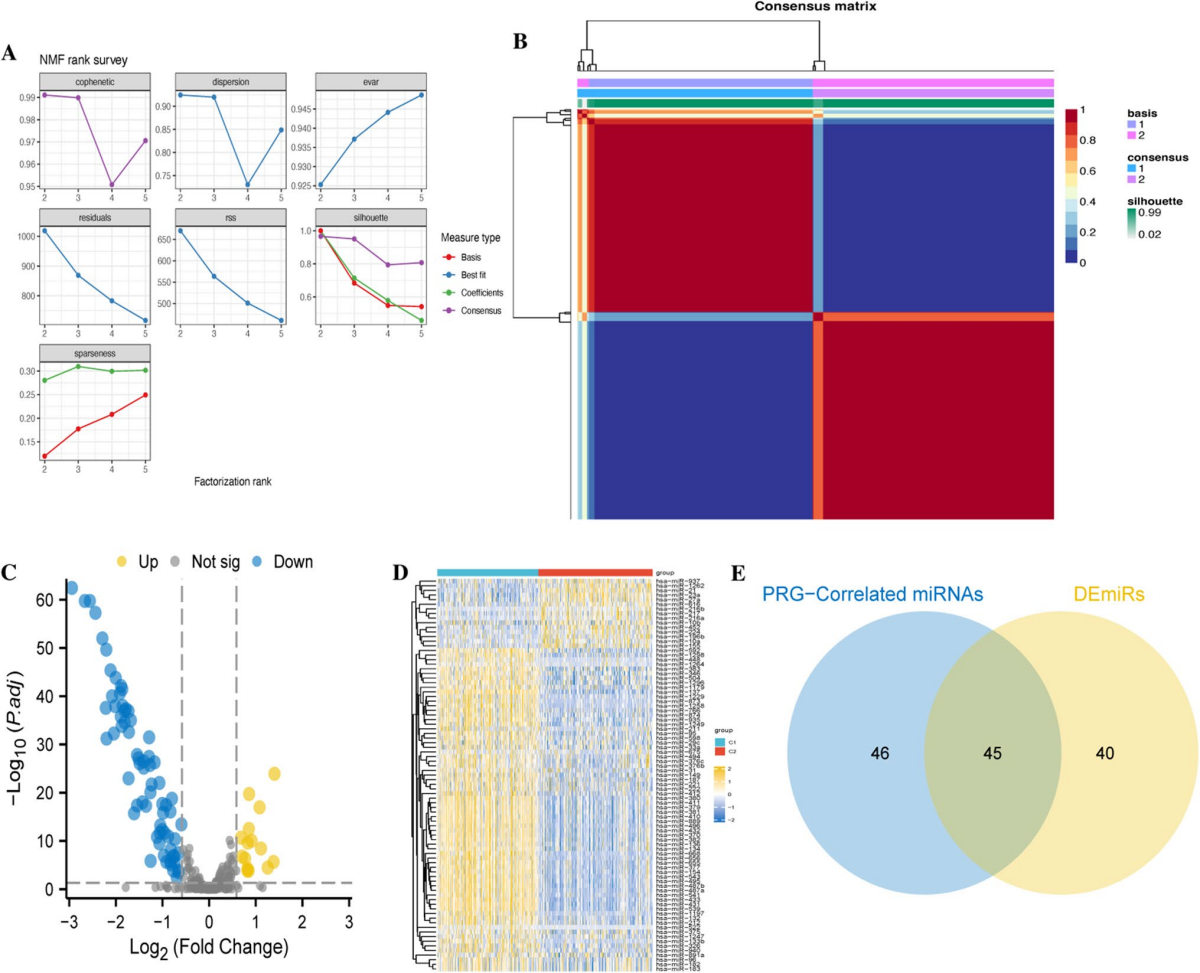
Classification of LGG Subtypes Based on miRNAs Significantly Associated with PRGs. **A** NMF classification parameter map; **B** Consensus clustering map from NMF clustering, distinctly categorizing LGG patients into two subtypes; **C** miRNAs differentially expressed (DEmiRs) between the two subtypes, yellow and blue plots indicate up- and down-regulated miRs in one isoform relative to the other, respectively; **D** Heatmap of expression for these DEmiRs, red and blue colors represent C2 and C1 subtypes, respectively. **E** Venn diagram identifying 45 miRNAs that show differential expression between the two subtypes and are highly correlated with PRG expression

To further explore the possible indirect correlations involved in miRNAs in LGG, 91 miRNAs were clustered using NMF, and LGG was classified into two subtypes, C1 and C2 (Fig. 3A, B), and 85 differentially expressed miRNAs (DEmiRs) existed between the two subtypes as shown in Fig. 3C, D. The predominant diagnosis in the C1 group was "Oligodendroglioma, NOS", whereas the diagnosis in the C2 group was "Astrocytoma, anaplastic", and the proportion of patients receiving treatment was higher in the C2 group, with a significant difference in the proportion of patients receiving treatment between the two groups (Supplementary Table 3). Forty-five of these DEmiRs remained highly correlated with PRGs expression, and these miRNAs may be involved in a complex regulatory network in LGG and are closely linked to PRGs (Fig. 3E).

### Construction of miRNA prediction model by lasso

**Fig. 4:**
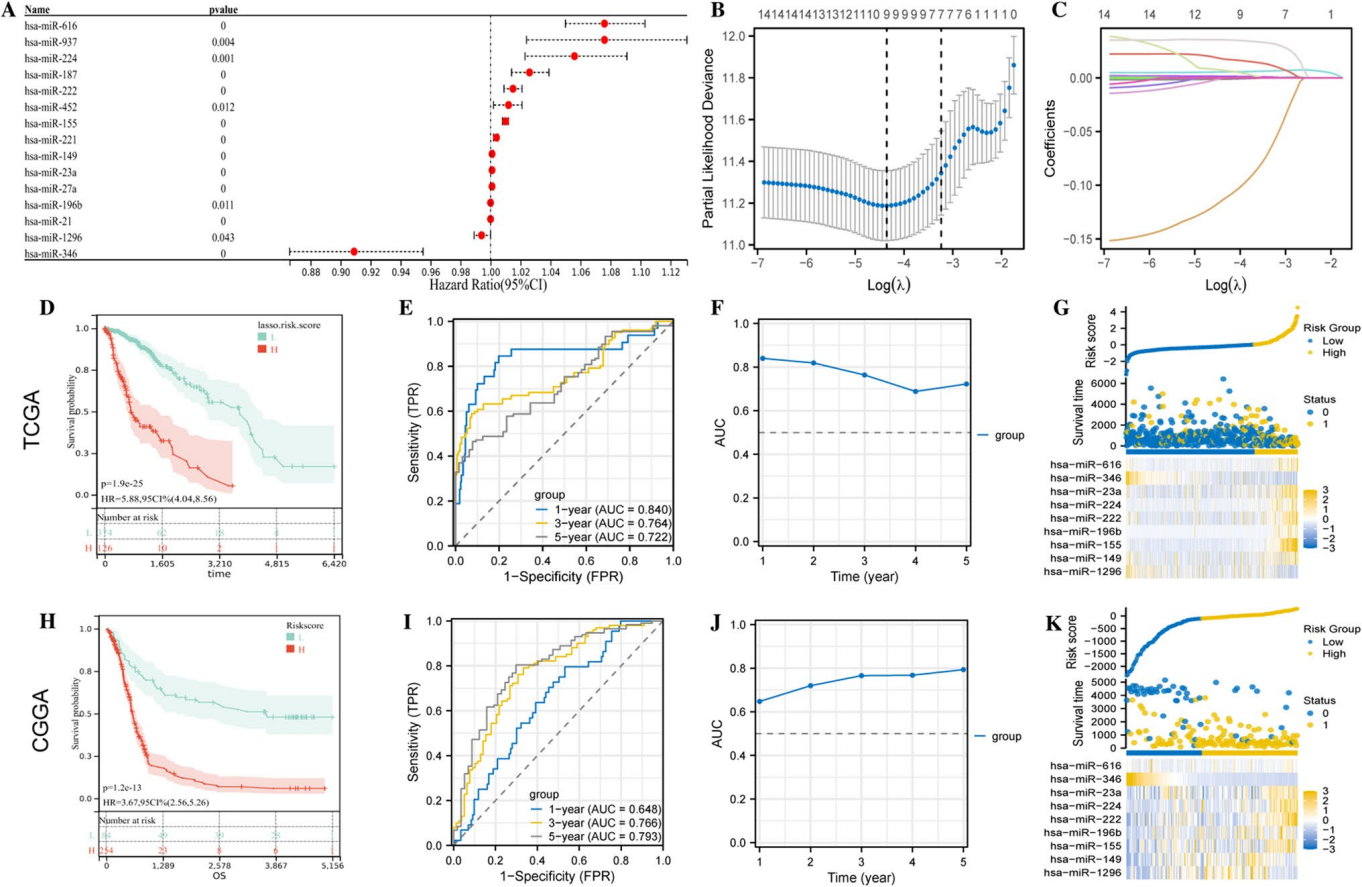
Construction of miRNA prediction model for prognosis of LGG patients using LASSO regression analysis. **A** Forest plot to show the 15 prognostically relevant miRNAs obtained from one-way Cox analysis; **B** Ten-fold cross-validation to select the best log(lamda) in the TCGA dataset; **C** Trajectory plot of lasso coefficients; **D** K-M survival curves of the high and low risk groups in the TCGA dataset; **E** Time dependent ROC; **F** AUC of the TCGA dataset; **G** Risk factor plots of the TCGA dataset; **H** K-M survival curves of the high- and low-risk groups in the CGGA dataset; **I** Time-dependent ROC of the CGGA dataset; **J** AUC of the CGGA dataset; **K** Risk factor plots of the CGGA dataset

To construct PRG-related miRNA prognostic features to predict the prognosis of LGG patients, univariate Cox regression analysis was first applied to screen miRNAs that were significantly associated with patients' overall survival (OS) (Fig. 4A). Subsequently, lasso further selected 15 prognostically relevant miRNAs. In total, 9 miRNAs and their corresponding coefficients were obtained (Fig. 4B, C), and the patient risk score was calculated using the following formula:

$$ {\text{Risk score}} = - 0.00116618 \times {\text{hsa}} - {\text{miR}} - 1296 + 0.001418971 \times {\text{hsa}} - {\text{miR}} - 149 + 0.00548326 \times {\text{hsa}} - {\text{miR}} - 155 + 0.000120955 \times {\text{hsa}} - {\text{miR}} - 196{\text{b}} + 0.017413605 \times {\text{hsa}} - {\text{miR}} - 222 + 0.006998872 \times {\text{hsa}} - {\text{miR}} - 224 + 3.43489{\text{E}} - 05 \times {\text{hsa}} - {\text{miR}} - 23{\text{a}} + - 0.112726538 \times {\text{hsa}} - {\text{miR}} - 346 + 0.035499089 \times {\text{hsa}} - {\text{miR}} - 616 $$

TCGA-miRNA expression profiles were used as the training set and CGGA-miRNA expression profiles as the validation set, and the optimal cut-off values were used to classify high-risk and low-risk groups. We evaluated the model with KM analysis, ROC curves, and risk factors to assess its prognostic potential in LGG patients. AUC areas greater than 0.6 at 1, 3, and 5 years were observed in both TCGA and CGGA for patients in the high-risk group (P < 0.05). It was found that the high-risk group had a significantly worse prognosis than the low-risk group, and the model expressed a more stable prognostic predictive ability (Fig. 4D–K).

### LGG patients in different risk groups present different clinical features

**Fig. 5:**
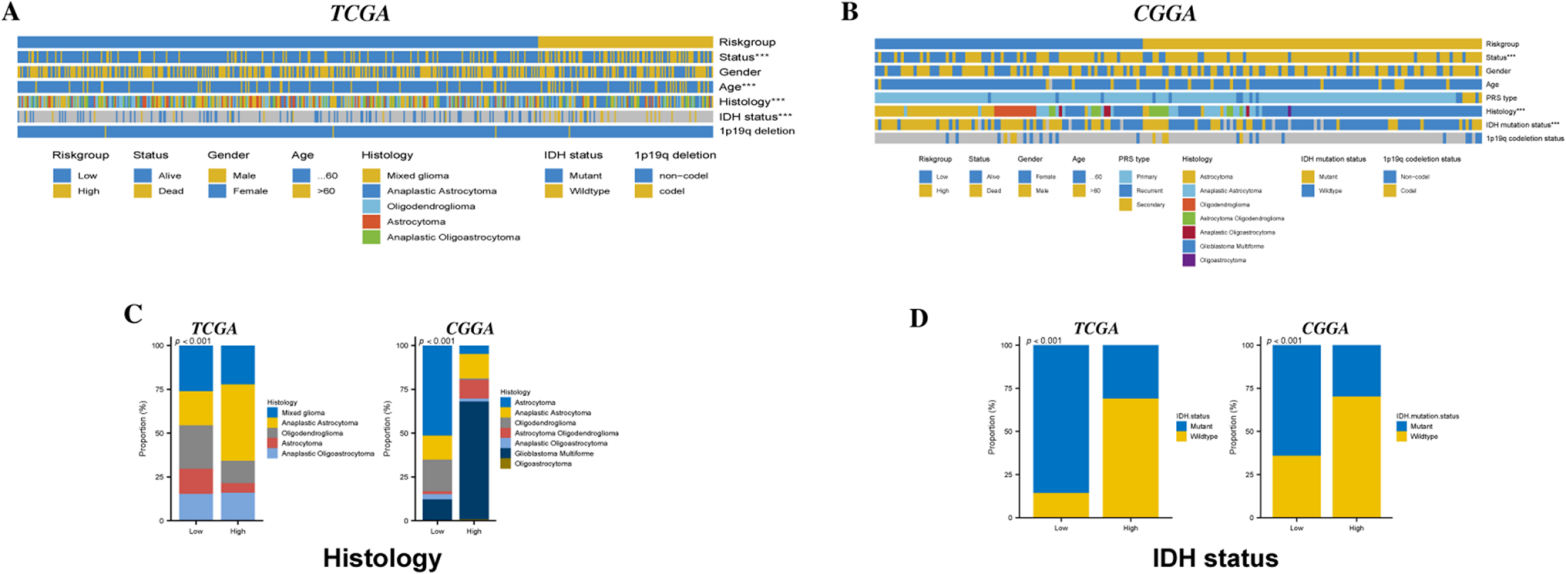
Differences in Risk Score and Clinical Characteristics between patients of different risk levels. Heatmaps presenting the distribution of clinical characteristics between patients grouped by different risk levels in the (**A**) TCGA and (**B**) CGGA databases; grouped stacked bar charts presenting the frequency of (**C**) Histology and (**D**) IDH mutation statuses in patients grouped by different risk levels in these two datasets

In-depth analysis of the clinicopathological features of these risk groups revealed that patients in the high-risk group died more frequently, with different histological stages and IDH mutations (Fig. 5A, B), and that high-risk patients had histologically higher rates of mixed gliomas and Anaplastic Astrocytoma, and that the proportion of IDH mutations was higher in low-risk LGG patients (Fig. 5C, D). Were higher (Fig. 5C, D). These differences reveal the importance of Risk scores in the prognosis of LGG patients and provide new perspectives for future clinical interventions.

### Nomogram construction and validation

**Fig. 6:**
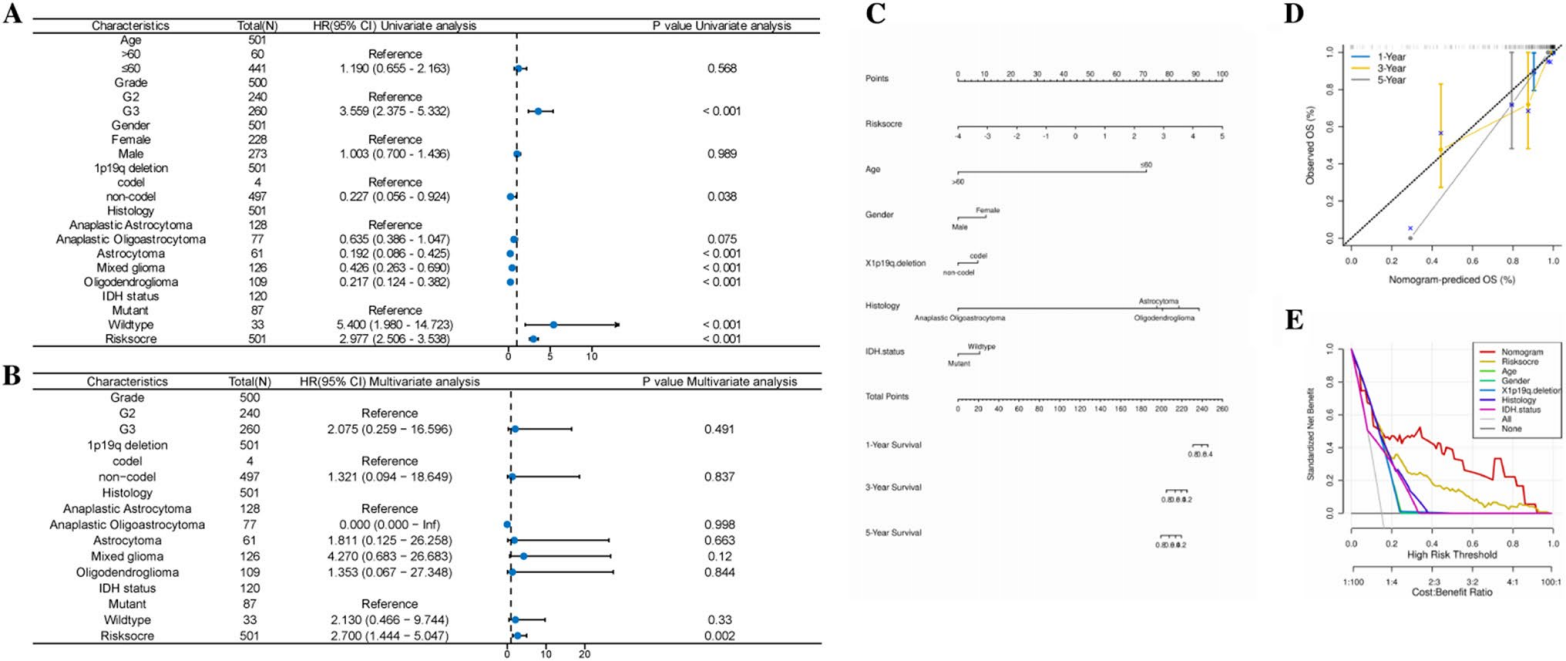
PMI-based construction of nomograms and evaluations. In LGG patients: **A** Univariate Cox analysis and (**B**) Multivariate Cox analysis; **C** construction of nomograms to predict prognosis; and (**D**) calibration curves

PMI scores were able to independently predict the prognosis of LGG patients both in univariate and multivariate Cox analyses (Fig. 6A, B). A Nomogram was constructed to predict the survival rate of LGG patients at 1,3,5 years (Fig. 6C), and the calibration curves (Fig. 6D) were used to assess the accurate predictive ability of the Nomogram for the prognosis, and the results of DCA indicated that the Nomogram was the best predictor of all the predictive factors optimally (Fig. 6E), a finding that suggests the potential of PMI-based construction of nomograms to provide valuable prognostic information for LGG patients.

### Differences in potential molecular mechanisms in LGG patients from different risk groups

**Fig. 7:**
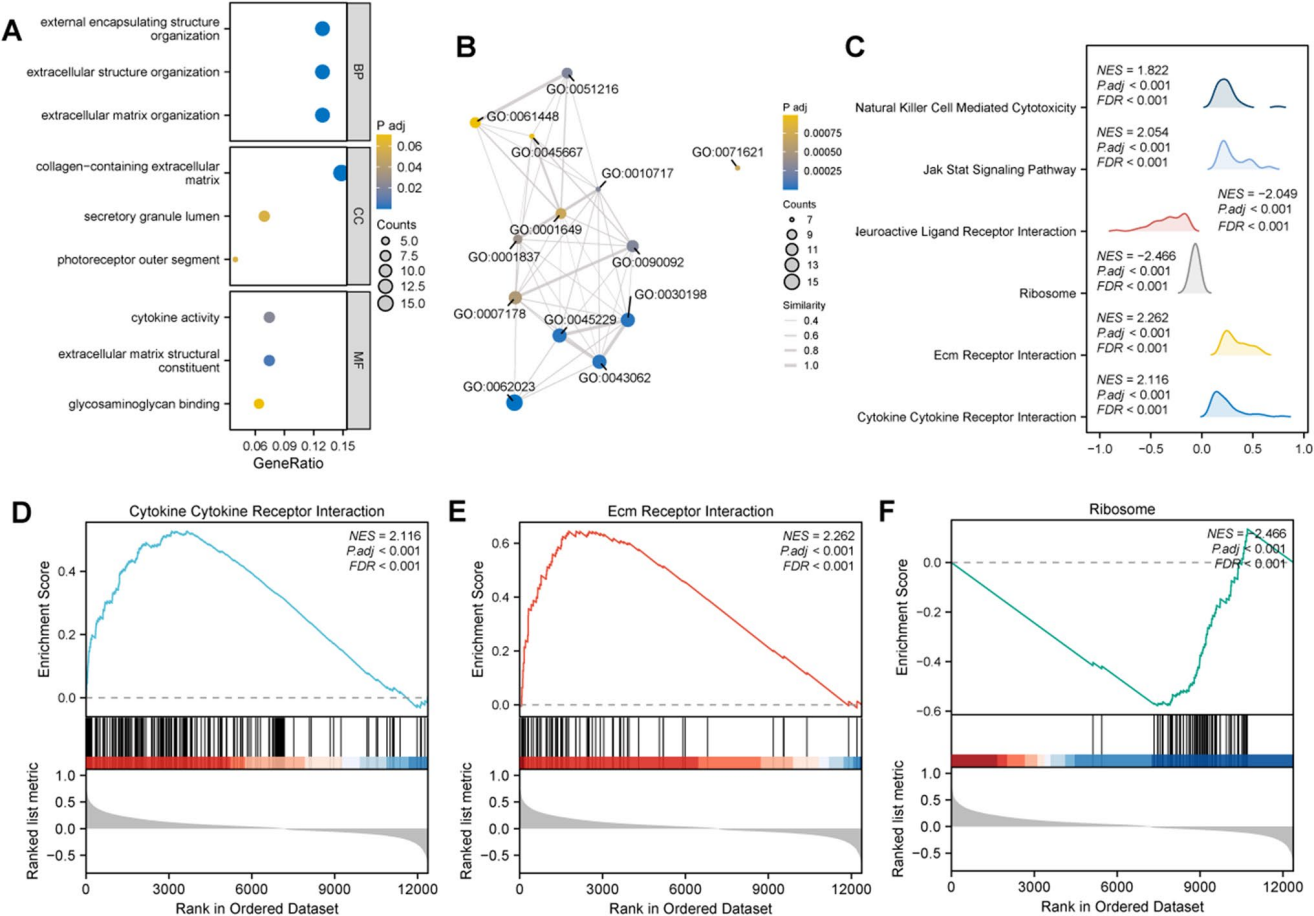
Functional enrichment analyses of high- and low-risk LGG groups. Differential genes between groups were analysed for (**A**) Gene ontology (GO) enrichment; **B** Emap plot showing pairwise similarity between significantly enriched terms; **C** Mountain range plot demonstrating significantly enriched KEGG pathways; **D** Cytokine-cytokine receptor interaction; **E** Ecm receptor interaction; **F** GSEA map of Ribosome. *BP* biological process, *CC* cellular component, *MF* molecular function

After enrichment analysis of differential genes in LGG patients in high and low-risk groups, we found that these genes were significantly enriched in key biological processes such as extracellular matrix organisation, extracellular structure organisation, external encapsulating structure organisation and other key biological processes were significantly enriched (Fig. 7A). Emap plots (Fig. 7B) revealed high similarity between the significantly enriched functions (adj P < 0.001), which may be involved in LGG developmental processes through interactions. In addition, GSEA identified significantly enriched pathways (Fig. 7C), and we found that the Cytokine-cytokine receptor interaction and Ecm receptor interaction pathway was significantly enriched in the high-risk group (Fig. 7D, E), which suggests the inflammatory response and extracellular matrix remodelling in the high-risk patients' importance. On the contrary, the Ribosome pathway was significantly enriched in the low-risk group (Fig. 7F), suggesting that the role of protein synthesis processes in maintaining normal cellular function may be associated with a better prognosis.

### Association of immune microenvironment heterogeneity with low-grade glioma risk score

**Fig. 8:**
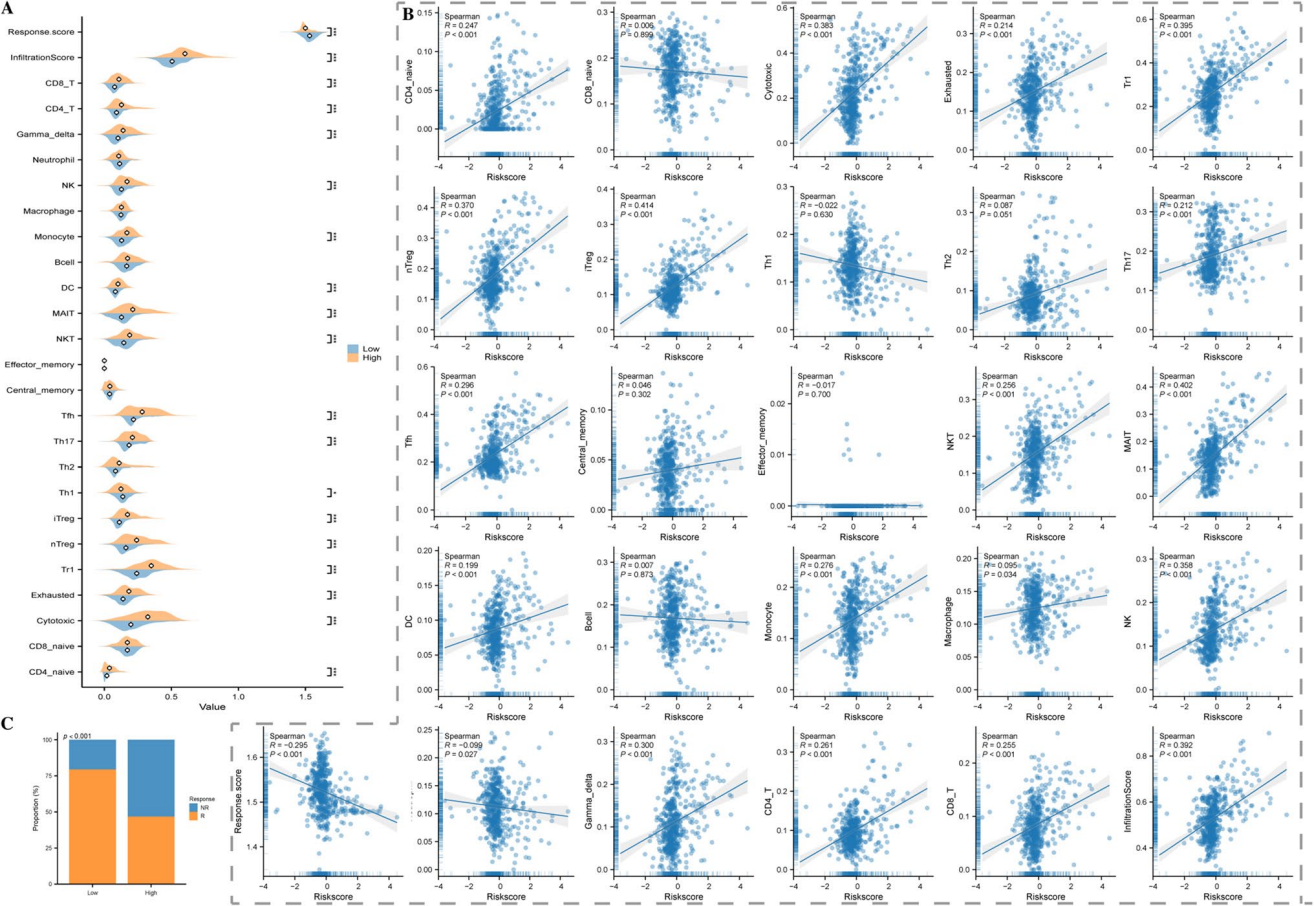
Correlation between immune microenvironment heterogeneity and risk scores for low-grade gliomas. **A** The proportions of 18 T and B cells, NK cells, monocyte cells, macrophage cells, neutrophil cells, and DC cells, and the corresponding immune scores; **B** Scatterplot of correlation of Riskscore with 24 immune cells; **C** Comparison of proportions of immune response in high and low risk groups

The proportion of different types of immune cells was significantly different between the high-risk and low-risk LGG groups (Fig. 8A). It was especially noticeable that the infiltration score for the high-risk group was significantly higher than for the low-risk group, while the response score for the high-risk group was significantly lower. In addition, correlation analysis with Riskscore revealed that immune cells, including CD4 native, iTreg, NK, and MAIT cells, showed a significant correlation with the risk score, whereas the Response score showed a significant inverse correlation (Fig. 8B). The proportion of immune response was even lower in the high-risk group of patients (Fig. 8C). This finding suggests that the tumour microenvironment of high-risk LGG patients may have more immunosuppressive properties, which may have contributed to the low responsiveness to immunotherapy.

### Potential drug candidates for LGG treatment

**Fig. 9:**
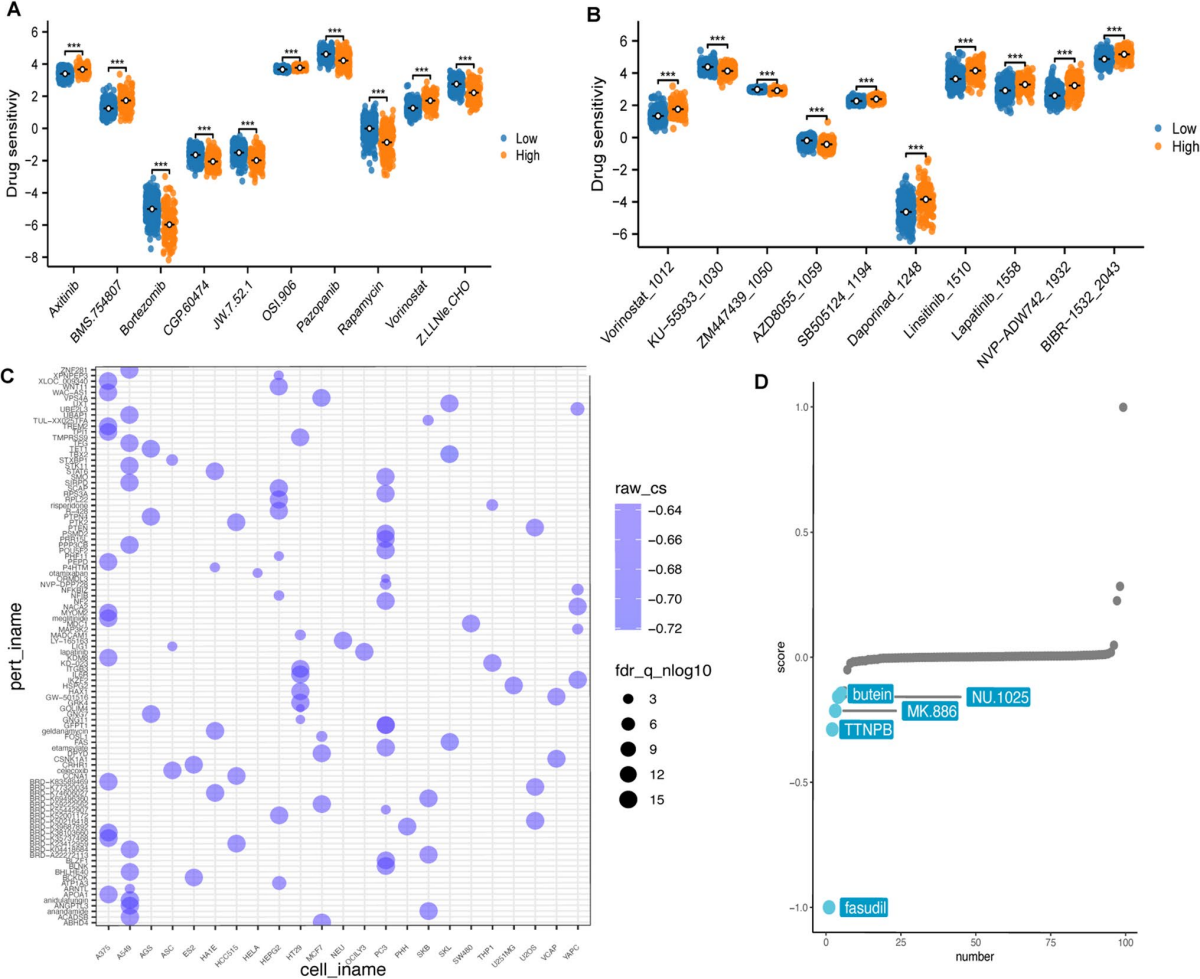
Comparison of drug sensitivity and drug prediction. Drug sensitivity of common drugs for LGG patients in high and low risk groups was assessed using (**A**) pRRophetic and (**B**) oncoPredicti; **C** relationship between molecularly targeted Therapy and common cell lines was assessed using CMap; and **D** predicted top 5 most likely therapeutic drugs

Drug sensitivity analyses showed significant differences in the name-sensitivity of some common drugs, such as Axitinib, Bortexomib, and Vorinostat, among others, in patients with different risk classes (Fig. 9A, B). Further CMap analyses showed that different compounds presented variable strengths of action in common tumour cell lines (Fig. 9C), and as shown in Fig. 9D, fasudil obtained the lowest combination score, implying that it possesses the potential to reverse the disease state of high-risk LGG patients, and is thus a promising candidate for the treatment of patients with poor prognosis LGG (see Fig. 9D). These findings are valuable in guiding personalised treatment regimens for LGG.

### Expression levels of 9 miRNAs and their prognostic profiles

**Fig. 10:**
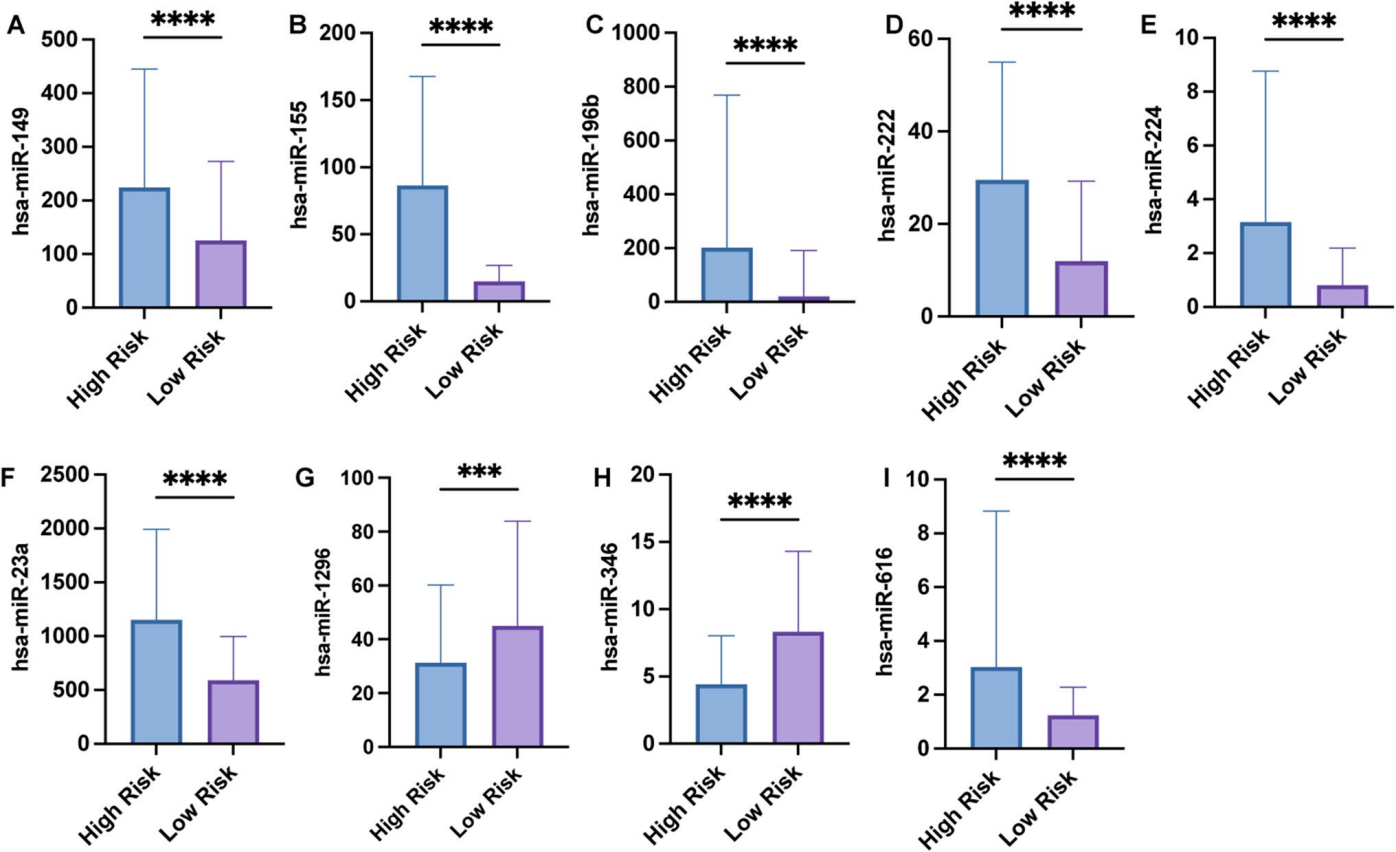
Expression levels of 9 miRNAs. patients in high and low risk groups (**A**) hsa-miR-149; **B** hsa-miR-155; **C** hsa-miR-196b; **D** hsa-miR-222; **E** hsa-miR-224; **F** hsa-miR-23a; **G** hsa-miR-1296; **H** hsa-miR-346; **I** hsa- miR-616’s RNA-seq expression levels of miR-616

**Fig. 11:**
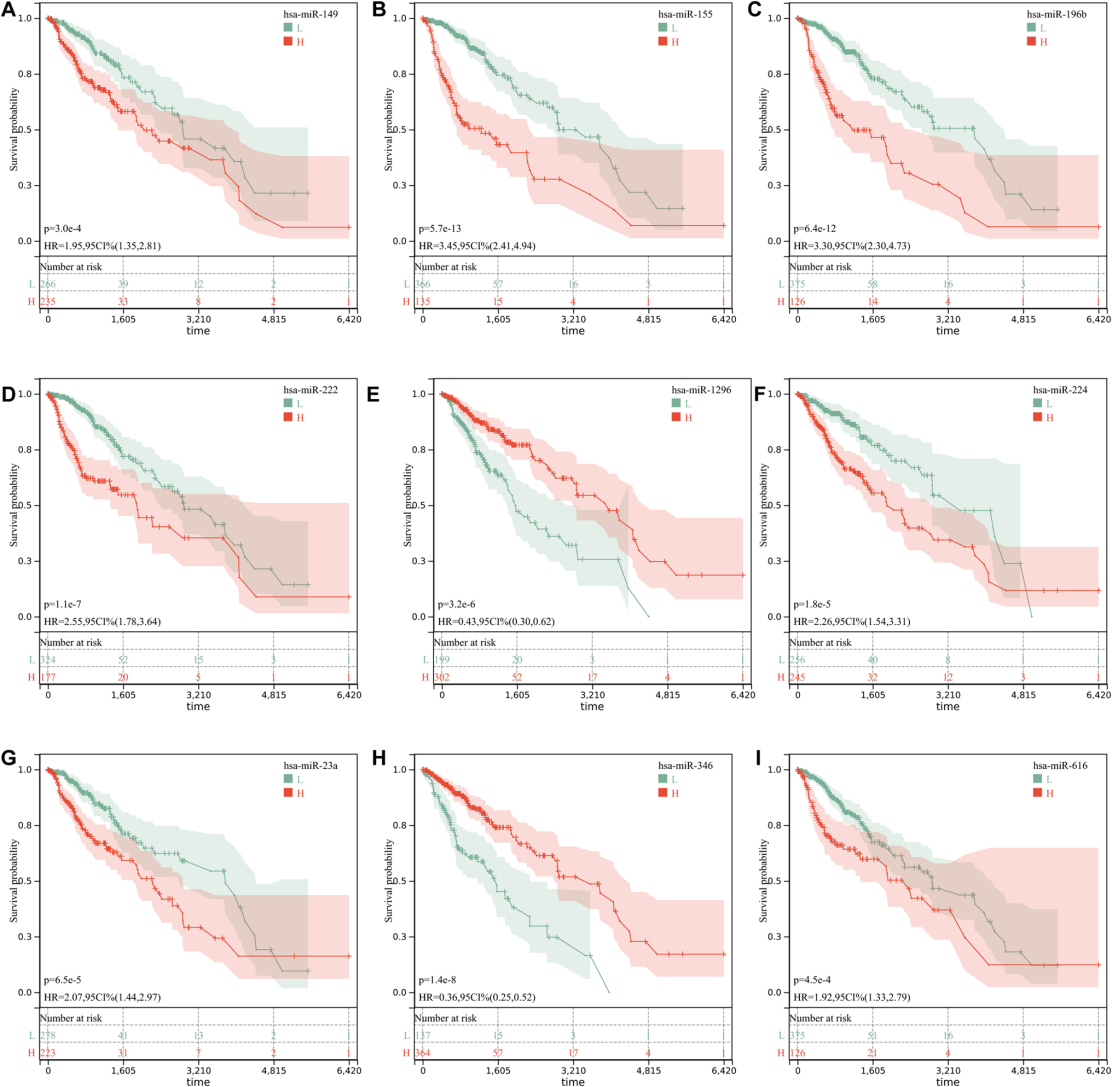
K-M survival curves of the 9 miRNAs. **A** hsa-miR-149; **B** hsa-miR-155; **C** hsa-miR-196b; **D** hsa-miR-222; **E** hsa-miR-1296; **F** hsa-miR-224; **G** hsa-miR-23a; **H** hsa-miR-346; **I** hsa-miR-616 high expression and low expression K-M survival curves of LGG patients

**Fig. 12:**
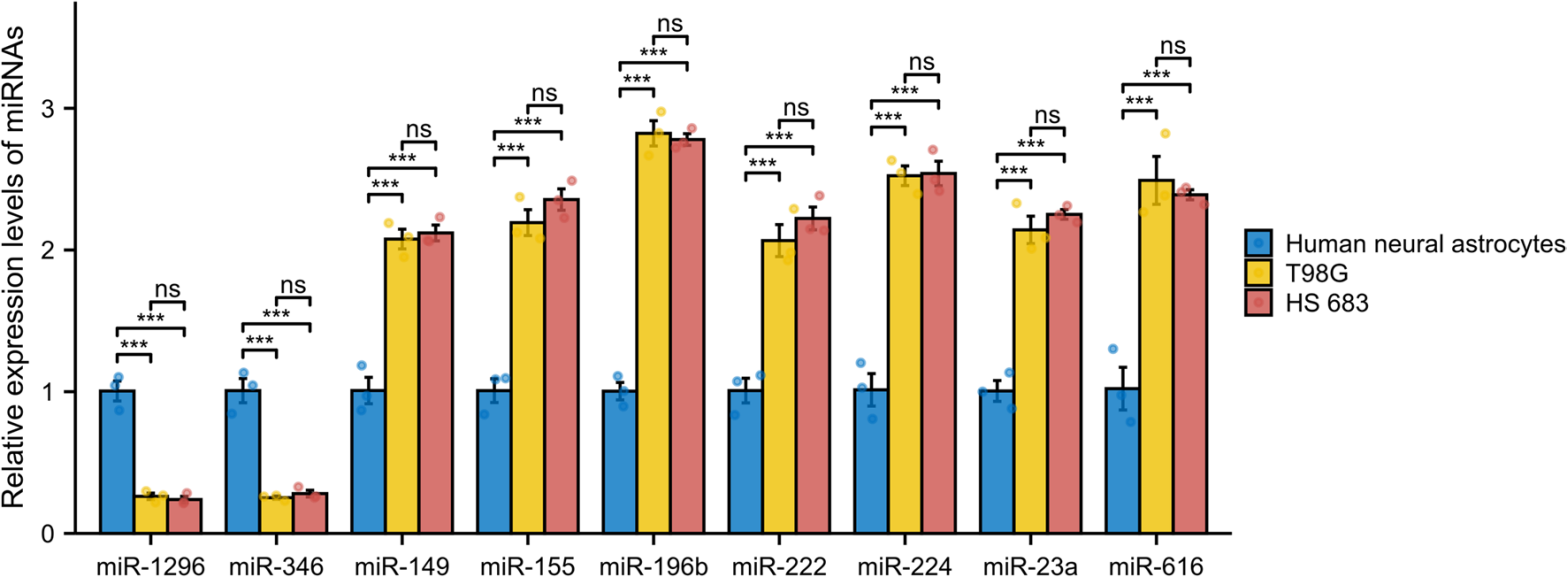
Expression of 9 miRNAs between Human neural astrocytes and glioma cells. Relative expression levels of miR-1296, miR-346, miR-149, miR-155, miR-222, miR-224, miR-23a, miR-616 in human neural astrocytes and T98G, HS 683 were detected using qPCR

We found that, as shown in Fig. 10, except for hsa-miR-1296 and hsa-miR-346, other miRNAs (hsa-miR-149, hsa-miR-155, hsa-miR-196b, hsa-miR-222, hsa-miR-224, hsa-miR-23a, hsa-miR- 616) were expressed at significantly higher levels in high-risk patients than in low-risk patients. Moreover, except for these two miRNAs, the other seven miRNAs could be pro-oncogenic, and patients with high expression levels had shorter survival, which could be used as a marker of poor prognosis in LGG patients (Fig. 11), while hsa-miR-1296 and hsa-miR-346 could be used as cancer-suppressing miRNAs, which might be important for maintaining normal cellular function and preventing malignant progression in LGG significance. Using the primer sequences in Table 2 for qPCR and finally normalised by U6, we found the same miRNA expression results in the cells (Fig. 12).

**Table 2:** Primer sequences for miRNA

		Sequence (5ʹ-3ʹ)	Melting temperature
miR-1296	Forward primer	CTTCGACCCTAACCCAGGTG	Tm = 59.4 ℃/20 nt
	Reverse primer	AGTGCAGGGTCCGAGGTATT	Tm = 58.5 ℃/20 nt
miR-346	Forward primer	TGTCTGCCCGCATGCCT	Tm = 61.0 ℃/17 nt
	Reverse primer	AGTGCAGGGTCCGAGGTATT	Tm = 58.5 ℃/20 nt
miR-149	Forward primer	GAGGGAGGGACGGGGG	Tm = 59.9 ℃/16 nt
	Reverse primer	AGTGCAGGGTCCGAGGTATT	Tm = 58.5 ℃/20 nt
miR-155	Forward primer	GCGCGCTCCTACATATTAGCA	Tm = 61.0 ℃/21 nt
	Reverse primer	AGTGCAGGGTCCGAGGTATT	Tm = 58.5 ℃/20 nt
miR-196b	Forward primer	GCGTCGACAGCACGACACT	Tm = 59.9 ℃/19 nt
	Reverse primer	AGTGCAGGGTCCGAGGTATT	Tm = 58.5 ℃/20 nt
miR-222	Forward primer	GCGCGAGCTACATCTGGCTA	Tm = 61.6 ℃/20 nt
	Reverse primer	AGTGCAGGGTCCGAGGTATT	Tm = 58.5 ℃/20 nt
miR-224	Forward primer	GCGAAAATGGTGCCCTAGTG	Tm = 60.6 ℃/20 nt
	Reverse primer	AGTGCAGGGTCCGAGGTATT	Tm = 58.5 ℃/20 nt
miR-23a	Forward primer	GCGATCACATTGCCAGGG	Tm = 60.0 ℃/18 nt
	Reverse primer	AGTGCAGGGTCCGAGGTATT	Tm = 58.5 ℃/20 nt
miR-616	Forward primer	CGCGAGTCATTGGAGGGTTT	Tm = 62.3 ℃/20 nt
	Reverse primer	AGTGCAGGGTCCGAGGTATT	Tm = 58.5 ℃/20 nt

## Discussion

Incorporating molecular diagnostics has become key to advancing precision medicine for LGG, particularly in the search for and application of effective markers, to provide more personalised therapeutic strategies and thereby improve patient survival [3, 19]. In this context, the role of Parthanatos-associated miRNAs in the development of LGG aroused our interest. Although Parthanatos and miRNAs have been heavily explored as a distinctive cell death modality and common diagnostic marker, respectively, in a variety of tumour types [3, 9, 20–22], few studies have combined the two, and their specific impact in LGG remains unclear. Therefore, this study aimed to investigate the expression pattern of Parthanatos-related miRNAs in LGG and their correlation with patient prognosis, to provide new strategies and directions for the treatment of LGG.

In this study, firstly, we verified the function and expression of the screened PRGs, and the results showed that PRGs were associated with oxidative stress response, which was consistent with previous studies and revealed the critical role of PRGs in regulating the neuropathological processes associated with oxidative stress, especially in promoting neuronal cell death associated with oxidative stress-induced DNA damage [23, 24]. PRGs are also highly correlated with signalling pathways of cell death such as Necroptosis and Apoptosis, which also flanks the relevance of these genes in cell death [20, 25].

Subsequently, we performed a systematic analysis of the key roles of Parthanatos-related miRNAs in expression, prognosis and immune response in LGGs. Ninety-one miRNAs were screened for significant correlation with PRGs by Spearman correlation analysis. By exploring the heterogeneous expression patterns in LGG, 45 miRNAs that were significantly differentially expressed in LGG and still highly correlated with the expression of PRGs were identified. A PMI consisting of 9 miRNAs was established by multiplex analyses to assess the prognosis of patients with LGG. The PMI showed significant prognostic predictive ability in the present study. High-risk scores were strongly associated with shorter overall survival, whereas low-risk scores predicted a relatively better survival prognosis. Moreover, PMI showed robust stability (AUC > 0.6) in different datasets, and PMI could be used as an independent prognostic factor to predict a patient’s prognosis, and the nomogram composed of PMI also demonstrated a very high prognostic potential. This finding not only highlights the important role of these miRNAs in the biological behaviour of LGG but also provides a potential approach to complement traditional clinical and pathological indicators to further optimize treatment strategies for LGG patients.

Furthermore, the significant enrichment of extracellular matrix organisation and inflammatory signalling pathways in the high-risk group revealed enhanced pathophysiological challenges that these patients may face, including increased inflammation and ECM remodelling, which may promote tumour progression and worsen the prognosis. Activation of PARP-1 plays a key role in the inflammatory response, in particular through the augmentation of the expression of pro-inflammatory genes and participation in the regulation of the function of immune cells [20], such as parthanatos induction in NK cells [26] and lymphocytes [27], which emphasises that LGG patients in the high-risk group exhibit significantly increased levels of inflammation, suggesting that these miRNAs may influence the immune environment and prognosis of patients by regulating PARP-1 activation and related inflammatory pathways. Although no direct evidence has been provided for a direct link between Parthanatos and extracellular matrix remodelling, session the importance of PARP-1 activation in the regulation of cell fate, and the fact that the process of ECM remodelling in tumour progression and inflammatory response is often accompanied by cell death and alterations in nuclear activity [28, 29], it can be speculated that Parthanatos-associated miRNAs may indirectly promote ECM remodelling by affecting these nuclear proteases and related cell death pathways.

Riskscore showed a positive correlation with the proportion of most immune cells, but the lack of LGG patients in the high-risk group showed a more immunosuppressive tumour microenvironment, which may be due to the activation of immune escape mechanisms, resulting in low responsiveness to immunotherapy in these patients. Therefore, restoring the activity and function of immune cells may provide an opportunity to improve the responsiveness and effectiveness of immunotherapy in LGG patients. Secondly, drug sensitivity analysis and CMap prediction revealed some potential therapeutic candidates, such as Fasudil, which showed better efficacy potential in high-risk group patients. Fasudil has multiple effects of attenuating inflammatory response, promoting DNA repair, inhibiting vasoconstriction, and its multiprotective ability in both diabetic stroke and radiation injury [30–32]. Fasudil is showing some serious promise as a go-to treatment for folks battling high-risk LGG. What's exciting is that fasudil might just be the key to stabilizing brain blood flow and easing those nasty pressure issues caused by tumors [31]. That means we could be looking at way better outcomes for patients.

By closely examining nine specific miRNAs, we have hit on some fascinating insights about their critical role in tumor biology—particularly for LGG—and their exciting potential as biomarkers and targets for new treatments. Here is the kicker: except for hsa-miR-1296 and hsa-miR-346, which seem to act as tumor progression stoppers, the higher expressions of the remaining seven miRNAs were linked to not-so-great survival outcomes, suggesting they might be egging cancer on. Researchers have found that hsa-miR-1296 and hsa-miR-346 reverse some pretty nasty traits in other cancers, offering hope for patients with these miRNAs cranked up to expect better outcomes [33, 34]. But, we've barely scratched the surface in understanding how other miRNAs play into LGG, pointing us toward a huge area ripe for research [16, 35, 36]. It is crucial to dive deeper to truly understand these miRNAs' biological roles in LGG and their potential for therapy. It’s not just about revealing LGG’s pathological progress; it's about setting the stage for innovative therapies that target these miRNAs directly.

Although our study revealed the important role of Parthanatos-related miRNAs in LGG, some limitations remain. For example, Fan et al. [21]. Showed that patients with high Parthanatos scores had a poorer prognosis and that the viability, proliferation, invasion, and migration of glioma cells were significantly inhibited by silencing the Parthanatos-related gene COL8A1. In addition, temozolomide acting on glioma cells can effectively inhibit the expression of COL8A1, thus improving the malignant characterization of the cells. Given this, we plan to extend the downstream functional validation of the nine identified miRNAs in future studies and deeply investigate their effects on the biological behavior and drug sensitivity of low-grade glioma cells. In addition, to improve the generalization of our findings, we also hope to further expand the clinical sample cohort. Future work will be devoted to collecting samples from different subtypes of low-grade glioma patients from different medical centers to enhance the representativeness and practical application value of the study.

In this investigation, the pivotal role of Parthanatos-associated miRNAs in delineating prognostic risks and modulating immune responses within the context of LGG was elucidated, marking Fasudil as a potential agent for ameliorating outcomes in high-risk patient cohorts. These findings have expanded our laying a cornerstone for the evolution of personalized, more efficacious therapeutic interventions. Future research is poised to delve into the precise mechanisms through which these miRNAs influence LGG's pathophysiology and treatment efficacy, with an emphasis on developing tailored therapeutic strategies that resonate with individual patient profiles.

### Supplementary Information

Supplementary Material 1 (PDF 13 KB)

Supplementary Material 2 (PDF 1748 KB)

Supplementary Material 3 (DOCX 23 KB)

Supplementary Material 4 (DOCX 19 KB)

Supplementary Material 5 (DOCX 20 KB)

## Data Availability

The dataset used in this research has been written in the Methods section, and the data generated in the research has been stored in Dataverse at the link: https://doi.org/10.7910/DVN/G2NPKY.

## References

[1] Rimmer B, Bolnykh I, Dutton L, Lewis J, Burns R, Gallagher P (2023). Health-related quality of life in adults with low-grade gliomas: a systematic review. Qual Life Res.

[2] Miller KD, Ostrom QT, Kruchko C, Patil N, Tihan T, Cioffi G (2021). Brain and other central nervous system tumor statistics, 2021. CA Cancer J Clin.

[3] Sahm F, Brandner S, Bertero L, Capper D, French PJ, Figarella-Branger D (2023). Molecular diagnostic tools for the World Health Organization (WHO) 2021 classification of gliomas, glioneuronal and neuronal tumors; an EANO guideline. Neuro Oncol.

[4] Toader C, Eva L, Costea D, Corlatescu AD, Covache-Busuioc RA, Bratu BG (2023). Low-grade gliomas: histological subtypes, molecular mechanisms, and treatment strategies. Brain Sci.

[5] Claus EB, Walsh KM, Wiencke JK, Molinaro AM, Wiemels JL, Schildkraut JM (2015). Survival and low-grade glioma: the emergence of genetic information. Neurosurg Focus.

[6] Chakrabarty S, LaMontagne P, Shimony J, Marcus DS, Sotiras A (2023). MRI-based classification of IDH mutation and 1p/19q codeletion status of gliomas using a 2.5 D hybrid multi-task convolutional neural network. Neuro-Oncol Adv.

[7] Ma W, Mei P (2023). SLC10A3 is a prognostic biomarker and involved in immune infiltration and programmed cell death in lower grade glioma. World Neurosurgery.

[8] Moujalled D, Strasser A, Liddell JR (2021). Molecular mechanisms of cell death in neurological diseases. Cell Death Differ.

[9] Zhou Y, Liu L, Tao S, Yao Y, Wang Y, Wei Q (2021). Parthanatos and its associated components: promising therapeutic targets for cancer. Pharmacol Res.

[10] Messikommer A, Maru B, Seipel K, Valk PJ, Theocharides AP, Pabst T, et al. Cancer prognosis according to parthanatos features. bioRxiv. 2021:2021.05. 24.445484.

[11] David KK, Andrabi SA, Dawson TM, Dawson VL (2009). Parthanatos, a messenger of death. Front Biosci.

[12] Wang X, Ge P (2020). Parthanatos in the pathogenesis of nervous system diseases. Neuroscience.

[13] Novakova J, Slaby O, Vyzula R, Michalek J (2009). MicroRNA involvement in glioblastoma pathogenesis. Biochem Biophys Res Commun.

[14] Tluli O, Al-Maadhadi M, Al-Khulaifi AA, Akomolafe AF, Al-Kuwari SY, Al-Khayarin R (2023). Exploring the role of microRNAs in glioma progression, prognosis, and therapeutic strategies. Cancers.

[15] Kanwal N, Al Samarrai OR, Al-Zaidi HMH, Mirzaei AR, Heidari MJ (2023). Comprehensive analysis of microRNA (miRNA) in cancer cells. Cell Mol Biomed Rep.

[16] Zhang JH, Hou R, Pan Y, Gao Y, Yang Y, Tian W (2020). A five-microRNA signature for individualized prognosis evaluation and radiotherapy guidance in patients with diffuse lower-grade glioma. J Cell Mol Med.

[17] Hussen BM, Hidayat HJ, Salihi A, Sabir DK, Taheri M, Ghafouri-Fard S (2021). MicroRNA: a signature for cancer progression. Biomed Pharmacother.

[18] Yang C, Zhang H, Chen M, Wang S, Qian R, Zhang L (2022). A survey of optimal strategy for signature-based drug repositioning and an application to liver cancer. Elife.

[19] van den Bent MJ, Geurts M, French PJ, Smits M, Capper D, Bromberg JE (2023). Primary brain tumours in adults. The Lancet.

[20] Huang P, Chen G, Jin W, Mao K, Wan H, He Y (2022). Molecular mechanisms of parthanatos and its role in diverse diseases. Int J Mol Sci.

[21] Fan S, Li H, Liu K (2024). Molecular prognostic of nine parthanatos death-related genes in glioma, particularly in COL8A1 identification. J Neurochem.

[22] Suszynska M, Machowska M, Fraszczyk E, Michalczyk M, Philips A, Galka-Marciniak P (2024). CMC: Cancer miRNA Census–a list of cancer-related miRNA genes. Nucleic Acids Res.

[23] Xu X, Sun B, Zhao C (2023). Poly (ADP-Ribose) polymerase 1 and parthanatos in neurological diseases: from pathogenesis to therapeutic opportunities. Neurobiol Dis.

[24] Jiang D, Yang X, Ge M, Hu H, Xu C, Wen S (2023). Zinc defends against Parthanatos and promotes functional recovery after spinal cord injury through SIRT3-mediated anti-oxidative stress and mitophagy. CNS Neurosci Ther.

[25] Ren K, Pei J, Guo Y, Jiao Y, Xing H, Xie Y (2023). Regulated necrosis pathways: a potential target for ischemic stroke. Burns Trauma.

[26] Hansson M, Asea A, Ersson U, Hermodsson S, Hellstrand K (1996). Induction of apoptosis in NK cells by monocyte-derived reactive oxygen metabolites. J Immunol.

[27] Rongvaux A, Galli M, Denanglaire S, Van Gool F, Dreze PL, Szpirer C (2008). Nicotinamide phosphoribosyl transferase/pre-B cell colony-enhancing factor/visfatin is required for lymphocyte development and cellular resistance to genotoxic stress. J Immunol.

[28] Frolova AS, Chepikova OE, Deviataikina AS, Solonkina AD, Zamyatnin AA (2023). New perspectives on the role of nuclear proteases in cell death pathways. Biology.

[29] Wang L-Y, Liu X-J, Li Q-Q, Zhu Y, Ren H-L, Song J-N (2023). The romantic history of signaling pathway discovery in cell death: an updated review. Mol Cell Biochem.

[30] Yao Y, Chen C, Cai Z, Liu G, Ding C, Lim D (2022). Screen identifies fasudil as a radioprotector on human fibroblasts. Toxicol Res.

[31] Masumoto N, Tanabe Y, Saito M, Nakayama K (2000). Attenuation of pressure-induced myogenic contraction and tyrosine phosphorylation by fasudil, a cerebral vasodilator, in rat cerebral artery. Br J Pharmacol.

[32] Liu J, Mu Z, Wang L, Wen R, Wang Y, Yang G (2019). Reduction of brain injury after stroke in hyperglycemic rats via fasudil pretreatment. J Shanghai Jiaotong Univ (Sci).

[33] Tu G, Peng W, Peng X, Zhao Z, Shi S, Cai Q (2023). hsa_circ_0000519 promotes the progression of lung adenocarcinoma through the hsa-miR-1296-5p/DARS axis. Am J Cancer Res.

[34] Xu C, Yuan B, He T, Ding B, Li S (2020). Prognostic values of YTHDF1 regulated negatively by mir-3436 in glioma. J Cell Mol Med.

[35] Zhang Q, Chen S, Zhen Y, Gao P, Zhang Z, Guo H (2022). Circular RNA circFGFR1 functions as an oncogene in glioblastoma cells through sponging to hsa-miR-224–5p. J Immunol Res.

[36] Litak J, Grajkowska W, Bogucki J, Kowalczyk P, Petniak A, Podkowiński A (2022). PD-L1/miR-155 interplay in pediatric high-grade glioma. Brain Sci.

