# Supplementary figures and images for: Elucidating the impact of parthanatos-related microRNAs on the tumoral immune microenvironment and clinical outcome in low-grade gliomas
Source: Discov Oncol. 2024 May 10;15:153. doi: 10.1007/s12672-024-01025-w (PMC11087408; doi:10.1007/s12672-024-01025-w)

TCGA

CGGA

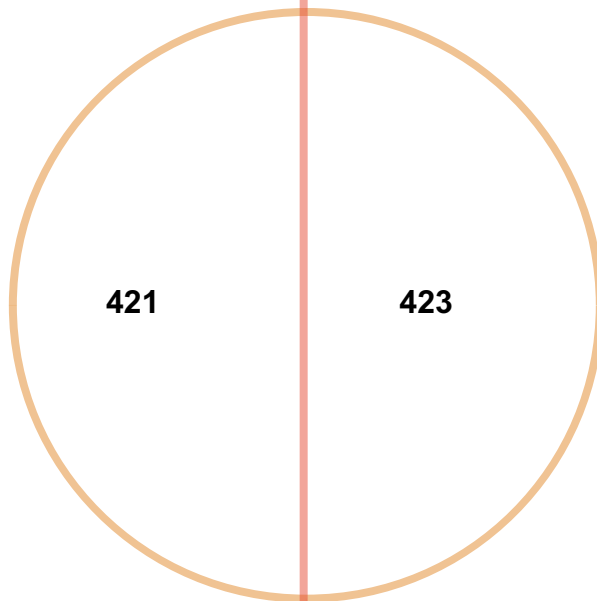

421

423

1453

Supplement: Supplementary file 1 — Supplementary Material 1 (PDF 13 KB) [file 12672_2024_1025_MOESM1_ESM.pdf]

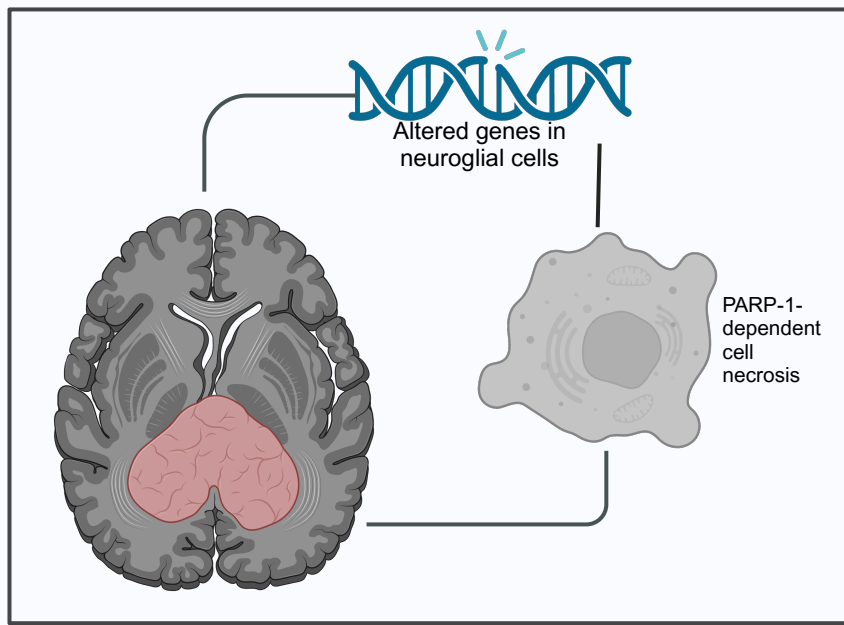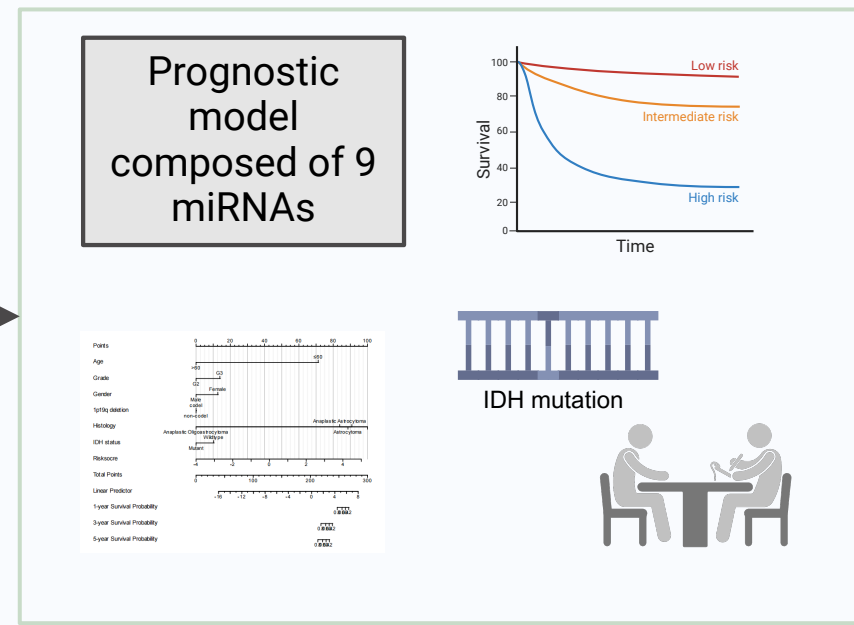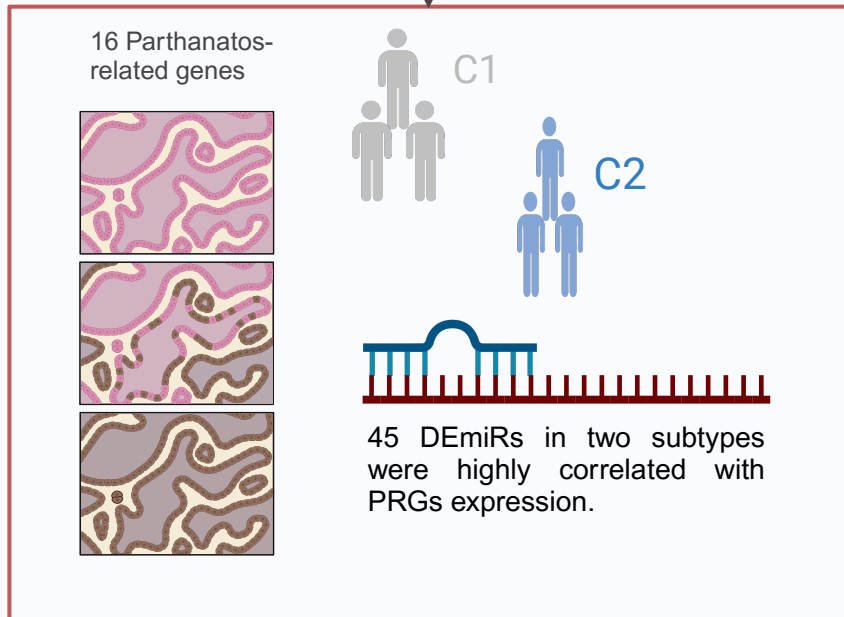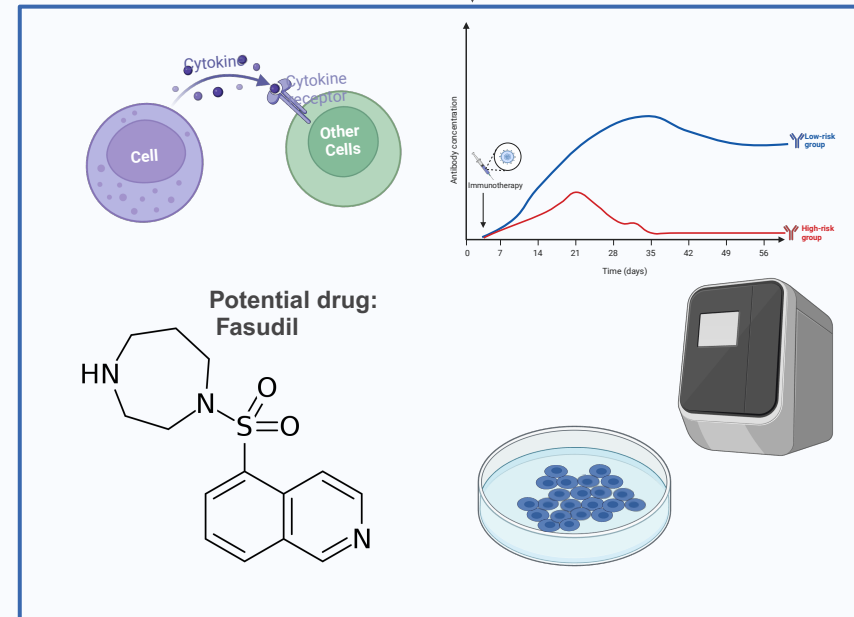

Supplement: Supplementary file 2 — Supplementary Material 2 (PDF 1748 KB) [file 12672_2024_1025_MOESM2_ESM.pdf]
