# Supplementary material for: Elucidating the impact of parthanatos-related microRNAs on the tumoral immune microenvironment and clinical outcome in low-grade gliomas
Source: Discov Oncol. 2024 May 10;15:153. doi: 10.1007/s12672-024-01025-w (PMC11087408; doi:10.1007/s12672-024-01025-w)
Supplement: Supplementary file 3 — Supplementary Material 3 (DOCX 23 KB) [file 12672_2024_1025_MOESM3_ESM.docx]

**Supplementary table 1** Summary of descriptive information for the TCGA dataset by "primary diagnosis "

|  | **Astrocytoma, anaplastic** | **Astrocytoma, NOS** | **Mixed glioma** | **Oligodendroglioma, anaplastic** | **Oligodendroglioma, NOS** | ***P*** |
| --- | --- | --- | --- | --- | --- | --- |
|  | ***N=128*** | ***N=61*** | ***N=126*** | ***N=76*** | ***N=106*** |  |
| Age | 44.7 (12.6) | 35.7 (10.5) | 41.2 (13.4) | 49.6 (13.9) | 43.1 (13.1) | <0.001 |
| Gender: |  |  |  |  |  | 0.991 |
| female | 59 (46.1%) | 26 (42.6%) | 58 (46.0%) | 34 (44.7%) | 47 (44.3%) |  |
| male | 69 (53.9%) | 35 (57.4%) | 68 (54.0%) | 42 (55.3%) | 59 (55.7%) |  |
| OS | 765 (785) | 1101 (1025) | 901 (850) | 861 (782) | 1208 (1209) | 0.004 |
| Staus: |  |  |  |  |  | . |
| Alive | 79 (61.7%) | 54 (88.5%) | 99 (78.6%) | 54 (71.1%) | 89 (84.0%) |  |
| Dead | 49 (38.3%) | 7 (11.5%) | 26 (20.6%) | 22 (28.9%) | 17 (16.0%) |  |
| Not Reported | 0 (0.00%) | 0 (0.00%) | 1 (0.79%) | 0 (0.00%) | 0 (0.00%) |  |
| Ethnicity: |  |  |  |  |  | . |
| hispanic or latino | 2 (1.56%) | 4 (6.56%) | 11 (8.73%) | 4 (5.26%) | 10 (9.43%) |  |
| not hispanic or latino | 117 (91.4%) | 47 (77.0%) | 111 (88.1%) | 69 (90.8%) | 90 (84.9%) |  |
| not reported | 9 (7.03%) | 10 (16.4%) | 4 (3.17%) | 3 (3.95%) | 6 (5.66%) |  |
| Race: |  |  |  |  |  | . |
| american indian or alaska native | 0 (0.00%) | 0 (0.00%) | 0 (0.00%) | 0 (0.00%) | 1 (0.94%) |  |
| asian | 1 (0.78%) | 1 (1.64%) | 1 (0.79%) | 2 (2.63%) | 2 (1.89%) |  |
| black or african american | 6 (4.69%) | 2 (3.28%) | 7 (5.56%) | 2 (2.63%) | 4 (3.77%) |  |
| not reported | 2 (1.56%) | 2 (3.28%) | 1 (0.79%) | 2 (2.63%) | 3 (2.83%) |  |
| white | 119 (93.0%) | 56 (91.8%) | 117 (92.9%) | 70 (92.1%) | 96 (90.6%) |  |
| prior_treatment: |  |  |  |  |  | 0.005 |
| No | 128 (100%) | 61 (100%) | 126 (100%) | 73 (96.1%) | 106 (100%) |  |
| Yes | 0 (0.00%) | 0 (0.00%) | 0 (0.00%) | 3 (3.95%) | 0 (0.00%) |  |
| site_of_resection_or_biopsy: |  |  |  |  |  | . |
| Brain, NOS | 20 (15.6%) | 6 (9.84%) | 13 (10.3%) | 7 (9.21%) | 13 (12.3%) |  |
| Cerebrum | 105 (82.0%) | 54 (88.5%) | 106 (84.1%) | 68 (89.5%) | 92 (86.8%) |  |
| Frontal lobe | 1 (0.78%) | 1 (1.64%) | 2 (1.59%) | 1 (1.32%) | 0 (0.00%) |  |
| Occipital lobe | 1 (0.78%) | 0 (0.00%) | 0 (0.00%) | 0 (0.00%) | 0 (0.00%) |  |
| Parietal lobe | 0 (0.00%) | 0 (0.00%) | 1 (0.79%) | 0 (0.00%) | 0 (0.00%) |  |
| Temporal lobe | 1 (0.78%) | 0 (0.00%) | 4 (3.17%) | 0 (0.00%) | 1 (0.94%) |  |
| synchronous_malignancy: |  |  |  |  |  | 0.565 |
| No | 127 (99.2%) | 60 (98.4%) | 123 (97.6%) | 74 (97.4%) | 106 (100%) |  |
| Not Reported | 1 (0.78%) | 1 (1.64%) | 2 (1.59%) | 2 (2.63%) | 0 (0.00%) |  |
| Yes | 0 (0.00%) | 0 (0.00%) | 1 (0.79%) | 0 (0.00%) | 0 (0.00%) |  |
| tissue_or_organ_of_origin: |  |  |  |  |  | . |
| Brain, NOS | 20 (15.6%) | 6 (9.84%) | 13 (10.3%) | 7 (9.21%) | 13 (12.3%) |  |
| Cerebrum | 105 (82.0%) | 54 (88.5%) | 106 (84.1%) | 68 (89.5%) | 92 (86.8%) |  |
| Frontal lobe | 1 (0.78%) | 1 (1.64%) | 2 (1.59%) | 1 (1.32%) | 0 (0.00%) |  |
| Occipital lobe | 1 (0.78%) | 0 (0.00%) | 0 (0.00%) | 0 (0.00%) | 0 (0.00%) |  |
| Parietal lobe | 0 (0.00%) | 0 (0.00%) | 1 (0.79%) | 0 (0.00%) | 0 (0.00%) |  |
| Temporal lobe | 1 (0.78%) | 0 (0.00%) | 4 (3.17%) | 0 (0.00%) | 1 (0.94%) |  |
| treatment_or_therapy: |  |  |  |  |  | . |
| no | 10 (7.81%) | 24 (39.3%) | 45 (35.7%) | 19 (25.0%) | 58 (54.7%) |  |
| not reported | 8 (6.25%) | 8 (13.1%) | 3 (2.38%) | 6 (7.89%) | 6 (5.66%) |  |
| yes | 110 (85.9%) | 29 (47.5%) | 78 (61.9%) | 51 (67.1%) | 42 (39.6%) |  |
