# Supplementary material for: Elucidating the impact of parthanatos-related microRNAs on the tumoral immune microenvironment and clinical outcome in low-grade gliomas
Source: Discov Oncol. 2024 May 10;15:153. doi: 10.1007/s12672-024-01025-w (PMC11087408; doi:10.1007/s12672-024-01025-w)
Supplement: Supplementary file 4 — Supplementary Material 4 (DOCX 19 KB) [file 12672_2024_1025_MOESM4_ESM.docx]

**Supplementary table 2** Summary of descriptive information for the CGGA dataset by "PRS_type "

|  | **Primary** | **Recurrent** | **Secondary** | ***P*** |
| --- | --- | --- | --- | --- |
|  | ***N=180*** | ***N=12*** | ***N=6*** |  |
| Grade: |  |  |  | 0.004 |
| WHO II | 59 (32.8%) | 1 (8.33%) | 0 (0.00%) |  |
| WHO III | 40 (22.2%) | 7 (58.3%) | 0 (0.00%) |  |
| WHO IV | 81 (45.0%) | 4 (33.3%) | 6 (100%) |  |
| Gender: |  |  |  | 0.614 |
| Female | 68 (37.8%) | 5 (41.7%) | 1 (16.7%) |  |
| Female | 1 (0.56%) | 0 (0.00%) | 0 (0.00%) |  |
| Male | 111 (61.7%) | 7 (58.3%) | 5 (83.3%) |  |
| Age | 42.4 (13.0) | 37.6 (10.2) | 33.7 (7.39) | 0.126 |
| OS | 1652 (1631) | 1166 (1245) | 714 (856) | 0.275 |
| Censor (alive=0; dead=1) | 0.70 (0.46) | 0.75 (0.45) | 0.80 (0.45) | 0.824 |
| Radio_status (treated=1;un-treated=0) | 0.88 (0.33) | 0.58 (0.51) | 0.33 (0.52) | <0.001 |
| Chemo_status (TMZ treated=1;un-treated=0) | 0.55 (0.50) | 0.83 (0.39) | 0.50 (0.55) | 0.151 |
| IDH_mutation_status: |  |  |  | 0.261 |
| Mutant | 70 (41.4%) | 7 (58.3%) | 4 (66.7%) |  |
| Wildtype | 99 (58.6%) | 5 (41.7%) | 2 (33.3%) |  |
| 1p19q_codeletion_status: |  |  |  | 0.601 |
| Codel | 5 (33.3%) | 2 (28.6%) | 0 (0.00%) |  |
| Non-codel | 10 (66.7%) | 5 (71.4%) | 4 (100%) |  |
| MGMTp_methylation_status: |  |  |  | 0.133 |
| methylation | 54 (32.0%) | 6 (54.5%) | 3 (60.0%) |  |
| un-methylated | 115 (68.0%) | 5 (45.5%) | 2 (40.0%) |  |
