# Supplementary material for: Elucidating the impact of parthanatos-related microRNAs on the tumoral immune microenvironment and clinical outcome in low-grade gliomas
Source: Discov Oncol. 2024 May 10;15:153. doi: 10.1007/s12672-024-01025-w (PMC11087408; doi:10.1007/s12672-024-01025-w)
Supplement: Supplementary file 5 — Supplementary Material 5 (DOCX 20 KB) [file 12672_2024_1025_MOESM5_ESM.docx]

**Supplementary table 3** Comparison of clinical characteristics of LGG subtypes delineated based on PRG significantly associated miRNAs

|  | **C1** | **C2** | ***P*** |
| --- | --- | --- | --- |
|  | ***N=232*** | ***N=261*** |  |
| Age | 43.5 (13.6) | 42.9 (13.2) | 0.615 |
| Gender: |  |  | 0.100 |
| female | 115 (49.6%) | 109 (41.8%) |  |
| male | 117 (50.4%) | 152 (58.2%) |  |
| OS | 963 (1013) | 943 (892) | 0.825 |
| Staus: |  |  | 0.006 |
| Alive | 189 (81.5%) | 184 (70.5%) |  |
| Dead | 43 (18.5%) | 76 (29.1%) |  |
| Not Reported | 0 (0.00%) | 1 (0.38%) |  |
| Ethnicity: |  |  | 0.172 |
| hispanic or latino | 17 (7.33%) | 14 (5.36%) |  |
| not hispanic or latino | 205 (88.4%) | 226 (86.6%) |  |
| not reported | 10 (4.31%) | 21 (8.05%) |  |
| Race: |  |  | 0.879 |
| american indian or alaska native | 0 (0.00%) | 1 (0.38%) |  |
| asian | 3 (1.29%) | 4 (1.53%) |  |
| black or african american | 10 (4.31%) | 10 (3.83%) |  |
| not reported | 6 (2.59%) | 4 (1.53%) |  |
| white | 213 (91.8%) | 242 (92.7%) |  |
| Primary_diagnosis: |  |  | <0.001 |
| Astrocytoma, anaplastic | 32 (13.8%) | 95 (36.4%) |  |
| Astrocytoma, NOS | 23 (9.91%) | 37 (14.2%) |  |
| Mixed glioma | 64 (27.6%) | 61 (23.4%) |  |
| Oligodendroglioma, anaplastic | 39 (16.8%) | 36 (13.8%) |  |
| Oligodendroglioma, NOS | 74 (31.9%) | 32 (12.3%) |  |
| prior_treatment: |  |  | 1.000 |
| No | 231 (99.6%) | 259 (99.2%) |  |
| Yes | 1 (0.43%) | 2 (0.77%) |  |
| site_of_resection_or_biopsy: |  |  | 0.116 |
| Brain, NOS | 21 (9.05%) | 37 (14.2%) |  |
| Cerebrum | 202 (87.1%) | 220 (84.3%) |  |
| Frontal lobe | 4 (1.72%) | 1 (0.38%) |  |
| Occipital lobe | 1 (0.43%) | 0 (0.00%) |  |
| Parietal lobe | 0 (0.00%) | 1 (0.38%) |  |
| Temporal lobe | 4 (1.72%) | 2 (0.77%) |  |
| synchronous_malignancy: |  |  | 0.066 |
| No | 226 (97.4%) | 260 (99.6%) |  |
| Not Reported | 5 (2.16%) | 1 (0.38%) |  |
| Yes | 1 (0.43%) | 0 (0.00%) |  |
| tissue_or_organ_of_origin: |  |  | 0.116 |
| Brain, NOS | 21 (9.05%) | 37 (14.2%) |  |
| Cerebrum | 202 (87.1%) | 220 (84.3%) |  |
| Frontal lobe | 4 (1.72%) | 1 (0.38%) |  |
| Occipital lobe | 1 (0.43%) | 0 (0.00%) |  |
| Parietal lobe | 0 (0.00%) | 1 (0.38%) |  |
| Temporal lobe | 4 (1.72%) | 2 (0.77%) |  |
| treatment_or_therapy: |  |  | 0.002 |
| no | 91 (39.2%) | 64 (24.5%) |  |
| not reported | 13 (5.60%) | 18 (6.90%) |  |
| yes | 128 (55.2%) | 179 (68.6%) |  |
